# Landscape scale ecology of *Tetracladium* spp. fungal root endophytes

**DOI:** 10.1186/s40793-022-00431-3

**Published:** 2022-07-25

**Authors:** Anna Lazar, Ryan M. Mushinski, Gary D. Bending

**Affiliations:** School of Life Sciences, The University of Warwick, Coventry, CV4 7AL UK

**Keywords:** *Tetracladium*, Aquatic hyphomycete, Endophyte, Root colonising, Agriculture, Diversity, Ecology

## Abstract

**Background:**

The genus *Tetracladium* De Wild. (Ascomycota) has been traditionally regarded as a group of Ingoldian fungi or aquatic hyphomycetes—a polyphyletic group of phylogenetically diverse fungi which grow on decaying leaves and plant litter in streams. Recent sequencing evidence has shown that *Tetracladium* spp. may also exist as root endophytes in terrestrial environments, and furthermore may have beneficial effects on the health and growth of their host. However, the diversity of *Tetracladium* spp. communities in terrestrial systems and the factors which shape their distribution are largely unknown.

**Results:**

Using a fungal community internal transcribed spacer amplicon dataset from 37 UK *Brassica napus* fields we found that soils contained diverse *Tetracladium* spp., most of which represent previously uncharacterised clades. The two most abundant operational taxonomic units (OTUs), related to previously described aquatic *T. furcatum* and *T. maxilliforme,* were enriched in roots relative to bulk and rhizosphere soil. For both taxa, relative abundance in roots, but not rhizosphere or bulk soil was correlated with *B. napus* yield. The relative abundance of *T. furcatum* and *T. maxilliforme* OTUs across compartments showed very similar responses with respect to agricultural management practices and soil characteristics. The factors shaping the relative abundance of OTUs homologous to *T. furcatum* and *T. maxilliforme* OTUs in roots were assessed using linear regression and structural equation modelling. Relative abundance of *T. maxilliforme* and *T. furcatum* in roots increased with pH, concentrations of phosphorus, and increased rotation frequency of oilseed rape. It decreased with increased soil water content, concentrations of extractable phosphorus, chromium, and iron.

**Conclusions:**

The genus *Tetracladium* as a root colonising endophyte is a diverse and widely distributed part of the oilseed rape microbiome that positively correlates to crop yield. The main drivers of its community composition are crop management practices and soil nutrients.

**Supplementary Information:**

The online version contains supplementary material available at 10.1186/s40793-022-00431-3.

## Background

Aquatic hyphomycetes or Ingoldian fungi are important decomposers in freshwater ecosystems [1]. Spores of these fungi were first described from running freshwater streams in the 1940s [2], with species classified according to morphology—primarily sigmoid or tetraradiate [3]. Sexual reproduction of these fungi has never been observed, and the members of this group don’t share common morphological or ecological characteristics [4]. However, the common conidial shape suggests convergent evolution, and may contribute to spore dispersal via improved anchoring to the substrate or higher buoyancy for better aquatic dispersal [5]. Recently, use of high-throughput sequencing has revealed the presence of aquatic hyphomycetes in fungal communities inhabiting soil and plants, although the ecological importance of these fungi in terrestrial habitats is unknown [6].

The genus *Tetracladium* is a group of common aquatic hyphomycete that was first described by de Wildeman in 1893 [7], and sits within the ascomycete class Leotiomycetes in the Han Clade 9/Stamnaria lineage/Vandijckellaceae clade as *incertae sedis* [8]*.* The genus name was coined in response to its distinct ~ 60 × 100 µm tetra formatted conidiospores which have a central axis with three radiating branches [9]. Since the initial description of *Tetracladium*, the genus has been found to be ubiquitous in aquatic environments [9–14]. The first terrestrial observations of *Tetracladium* were from forest litter [11, 15, 16], with fungal spores detected in the water film covering fallen leaves [15]. However, most reports of *Tetracladium* in terrestrial environments came after the turn of the century as DNA sequencing techniques became more easily accessible. Most of this data comes from environmental metabarcoding studies, and there are only a few instances of *Tetracladium* ssp. being isolated in pure cultures. It has been hypothesised that there may have been under-reporting of *Tetracladium* in terrestrial habitats before the 2000s because of the strange nature of finding an aquatic organism in a terrestrial environment [6]. It is unclear whether the species described based on spore morphology from aquatic habitats and the DNA sequences identified from terrestrial environmental samples belong the same organisms. However, nuclear ribosomal internal transcribed spacer (ITS) amplicon analysis has shown no sequence-based differences between aquatic and terrestrial strains of a number of species, indicating that some species may have diverse ecological functions [6].

One of the first observations of plant endophytic *Tetracladium* sp. came from riparian plant roots [17]. These fungi do not appear to show host or habitat specificity as plant endophytes, and they have been found in roots of monocot species within Asparagales [18–20], Liliales [21], and Poales [22–24], as well as dicot species within Ericales [25], Brassicales [26, 27] and Vitales [28]. Furthermore, they have been found associated with Equisetaceae [29, 30] and Bryophytes [31–34]. *Tetracladium* has most frequently been described in metabarcoding studies of soil from disturbed agricultural and grassland habitats [35–38]. There are numerous reports of the genus from the Arctics [39–42] and they have also been found in unvegetated habitats including glaciers and bedrock [43–45].

The dual ecology of *Tetracladium* sp., and particularly their importance as plant endophytes, are still debated. Anderson et al. [9] investigated an aquatic *T*. *marchalianum* population over time and space and found that the fungus maintains a high genotypic diversity throughout the year. They suggested that this could be attributed to their alternative lifestyles as terrestrial plant endophytes [9]. It was proposed by Selosse et al. [6] that the terrestrial occurrence of aquatic hyphomycetes, and more specifically their endophytic nature, is attributed to the fungi precolonising plant tissues and building biomass, so in the event of abscission, they are already occupying the niche, and ready to decompose plant litter which reaches freshwater. It was also suggested that the tetraradiate spore morphology could aid them in becoming airborne [6]. Based on this theory *Tetracladium* sp. should be the most common in aerial plant tissues, however there is currently no evidence to suggest that this is true.

There is conflicting evidence on whether *Tetracladium* infection provides benefits to the host. Glasshouse experiments have shown that inoculation with *Tetracladium* sp. can have beneficial effects on plant growth [46], while other studies have shown no effects [47]. Importantly, Hilton et al. [38] found that *Tetracladium* sp. had a co-exclusion relationship with root pathogenic fungi, and relative abundance in roots was positively associated with crop yield. Furthermore, *T. marchalianum* showed an antagonistic effect against bacterial plant pathogens including *Erwinia chrysanthemi and Xanthomonas phaseoli,* although other *Tetracladium sp.* showed no such effects [48].

To date there have been no systematic studies that have investigated the diversity and distribution of *Tetracladium* in terrestrial habitats, and as a result the factors which shape *Tetracladium* spp. communities. Thus, the extent to which they interact with plants are unclear. In the current study we build on our earlier work [38] which characterised root fungal communities of *Brassica napus* across 37 UK fields to investigate (1) the diversity of *Tetracladium* spp. in soil and roots at the landscape scale (2) the extent to which different *Tetracladium* spp. are selectively recruited into roots and rhizosphere soil from bulk soil (3) the relationships between root, rhizosphere and soil populations of different *Tetracladium* spp. and crop yield and (4) to determine the importance of, and interactions between, soil nutrients, climate, soil physical properties, and crop management practices as drivers for the colonisation of roots by *Tetracladium* spp.

## Methods

### Sample collection and analyses

Root, rhizosphere soil and bulk soil samples were collected in March 2015 from 37 oilseed rape (*B. napus*) fields from 25 commercial farms in the UK. Five composite samples were taken from each field site. Loosely adhering soil was removed from the roots only leaving 2 mm rhizosphere soil. Roots with closely adhering soil were washed four times in sterile distilled water to release the rhizosphere soil which was then centrifuged (3250×*g* for 10 min) to leave a pellet of rhizosphere soil. Washed roots that were less than 2 mm diameter were cut into 5 mm pieces to obtain root samples. Bulk soil samples were sieved (7 mm followed by 2 mm sieve) then approximately 6 g was washed in sterile distilled water using the same washing method as the rhizosphere soil samples to ensure comparability. Following DNA extraction (PowerSoil-htp™ 96 Well Soil DNA Isolation Kit (MoBio Laboratories, Carlsbad, CA, USA)), the fungal community was amplified using internal transcribed spacer (ITS) primers fITS7-ITS4 [49]. Sequencing was performed with Illumina MiSeq technology (Illumina MiSeq Reagent Kit v3), and taxonomy assigned using Quantitative Insights into Microbial Ecology (QIIME 1.8) [50] with the UNITE ITS database [51]. Sequences were clustered to operational taxonomic units (OTU) [52] at 97% minimum identity threshold, and those OTUs assigned as *Tetracladium* spp. were selected for use in the current study. Metadata collected from each field included soil physico-chemical parameters (including C, N, P, micronutrient, pH, and soil type), climatic data, crop variety, rotation sequence and grain yield at the subsequent harvest. Full methodological details for sample preparation, DNA extraction, sequencing and bioinformatic analysis can be found in Hilton et al. [38]. Information about farm locations, *Tetracladium* spp. OTUs and metadata can be found in Additional file 4 A and B.

### Phylogenetic analyses

To obtain more detailed information about the phylogenetic relatedness of recovered *Tetracladium* spp. sequences, the most closely related sequences to these OTUs were downloaded, including two representative ITS2 sequences from all described species. Sequences were accessed from the NCBI GenBank, and were aligned with our *Tetracladium* OTU sequences using MAFFT v.7 (e-ins-I algorithm) [53]. The multiple sequence alignment can be found in Additional file 7. Maximum likelihood analyses were performed with RAxML on the CIPRES Science Gateway to build a phylogenetic tree using the default setting with 1000 bootstrap replicates [54, 55].

### Statistical analyses

Observed species counts were used to generate richness plots to study OTU abundance differences in the three sampled compartments (bulk soil, rhizosphere, and root) using *vegan* (version 2.6–2) in R (version 4.12) [56]. Rarefaction curves were constructed to assess the extent to which *Tetracladium* spp. richness was captured at the sequencing depth used. A heatmap was created to investigate the distribution of *Tetracladium* spp. OTUs across the sampling locations and between the soil, rhizosphere, and root compartments. Heatmap and rarefaction analyses were carried out using *phyloseq* (version 1.36.0) [57]. Significance of differences in taxa richness between compartments and differences in OTU relative abundance between crop genotypes and previous cultivated crops were tested using the Kruskal–Wallis rank sum test. *P* values were corrected for multiple comparisons with a Dunn’s test using the false discovery rate with the Benjamini–Hochberg method. Linear regression was used to correlate relative abundance to yield and rotation. Zero values were introduced to accommodate for fields that never had oilseed rape sown before, therefore rotation length values are reciprocal. Ternary plots were created using *ggtern* (version 3.3.5) [58] to understand the compartment preference of the OTUs.

Out of all the OTUs found that resembled the genus *Tetracladium* we chose the two most abundant OTUs, which were also substantively enriched within the root compartment, for detailed ecological analyses. Data was normalised using modified Z-scores. To test for drivers of relative abundance in the roots, we created a piecewise structural equation model (PSEM). First, a correlogram was created to better understand relationships between metadata and OTU relative abundance (Additional file 1) using base R functions [59]. Then, individual linear mixed-effect models were fitted with sampling location and soil compartment as random variables. Significant soil nutrient factors, relative to OTU abundance, taken from the correlogram output and the initial fitted model with all soil nutrients (modM) (Additional files 1 and 2), soil structure and climate were used as composite fixed variables. These composite variables were created so complicated constructs can be processed as simpler blocks that are easier to present and discuss [60]. Variable reduction was done to unmask significant relationships that may be missed if too many factors were included based on the LMM fitted with all variables. Individual models were fitted using the R package *lme4* [61]. Fixed variables were reduced via assessing best model fit using the *performance* package [62]. Finally, path models were fitted with the *piecewiseSEM* package [63], based on the findings of the individual models.

## Results

**Fig. 1:**
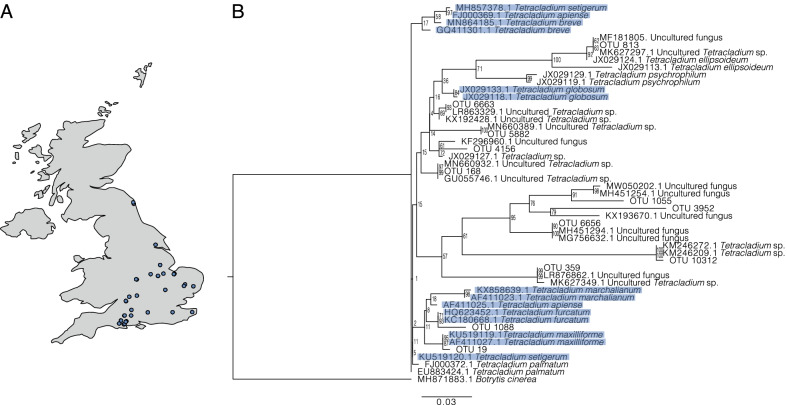
**A** Location of the sampling sites used in this study. **B** ITS sequence based maximum likelihood tree with posterior probability values of the twelve *Tetracladium* sp. OTUs and reference sequences. *Botrytis cinerea* was used as an outgroup. The scale bar denotes the number of nucleotide differences per site. Taxa highlighted in blue are traditionally considered aquatic

**Fig. 2:**
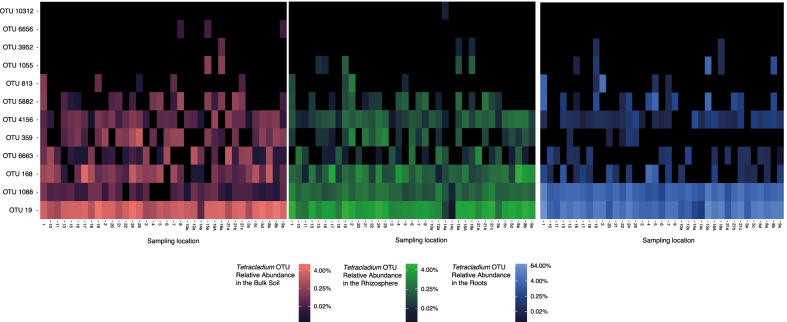
The distribution of *Tetracladium* sp. OTU relative abundance across the sampling locations in the bulk soil, the rhizosphere, and the roots

Across the 37 fields we found twelve OTUs that represented the genus *Tetracladium* (Fig. 1A). The closest match of the *Tetracladium* sequences to UNITE DOI is shown in Additional File 4 E. The accession numbers of the *Tetracladium* OTU sequences are shown in Additional File 4 F. Sequencing depth was sufficient to capture richness for most samples (Additional file 3). Higher abundance *Tetracladium* sp. OTUs (OTUs 19, 1088, 168, 6663, 359, 4156, and 5882) were found in all sampled fields, while lower abundance OTUs (813, 1055, 3952, 6656, and 10312) were found sporadically across sampling locations (Fig. 2, Additional file 4 C). The highest abundance OTUs, OTU 19 and 1088, grouped with *T*. *maxilliforme* and *T. furcatum* respectively, both of which have been described from water (Fig. 1B). OTU 813 clustered closely with, but was not identical to the species *T. elipsoideum*, which has been described from Arctic soil [44] and the closest uncultured environmental sequences to this OTU (MF181805.1 and MK627297.1) also originate from soil. OTUs 6663, 5882, 4156, and 168 clustered with uncultured *Tetracladium* sp. sequences in a clade close to *T. ellipsoideum*, *T. psychrophilum* and *T. globosum*. The closet uncultured environmental sequences for OTU 6663 (LR863329.1 and KX192428.1), OTU 5882 (MN660389.1), OTU 4156 (KF296960.1 and JX029127.1), and OTU 168 (GU055746.1) all originate from terrestrial samples. Environmental sequence MN660932.1—found in water—was a close match to OTU 168. OTUs 1055, 3952, 6656, 10312, and 359 formed a distinct clade with uncultured *Tetracladium* sp. sequences (MW050202.1, MH451254.1, KX193670.1, MH451294.1, MG756632.1, KM246272.1, MK246209.1, LR876862.1, and MK627349.1) found on land, likely representing uncharacterised species.

**Fig. 3:**
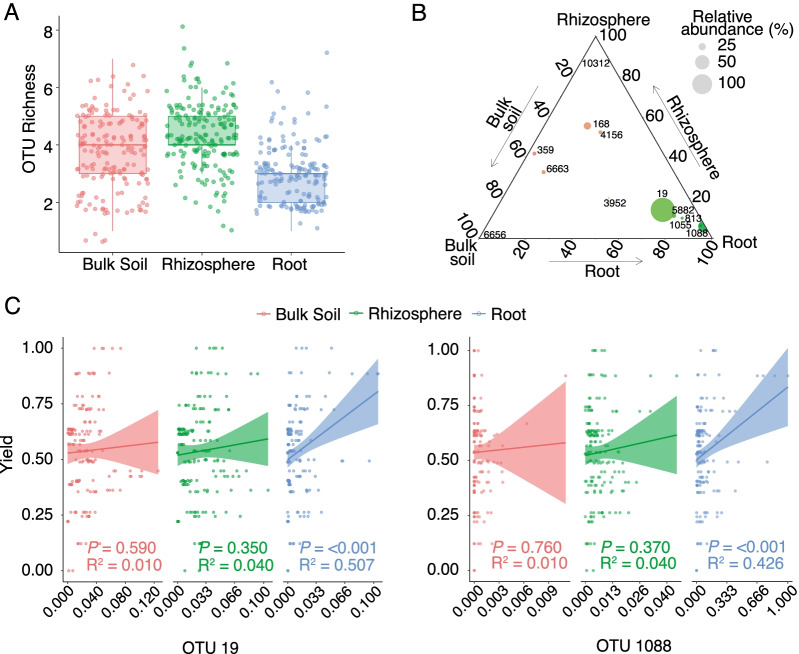
Analyses of *Tetracladium* OTU distribution in the bulk soil, the rhizosphere, and the roots. **A** Observed OTU richness in the three sampled compartments. Error bars represent standard error. **B** Ternary plots of *Tetracladium* OTU distribution across compartments. **C** Linear regression line fitted between OTUs and yield across the three compartments with significance values. OTU relative abundances and yield are normalised. The shaded region represents the 95% confidence limits for the estimated prediction

There was a significant difference between observed OTU richness of the three compartments (Fig. 3A), which was the lowest in the roots while the bulk soil and rhizosphere showed the same OTU richness (rhizosphere-bulk soil *P* =  < 0.001, root-bulk soil *P* =  < 0.001, root-rhizosphere *P* =  < 0.001). Five of the OTUs (OTUs 19, 5882, 1055, 813, and 1088) were the most abundant in the roots, while OTU 6656 was found in greater abundance in the bulk soil, and OTU 10312 was only found in the rhizosphere. The rest of the OTUs did not show a specific preference for compartment (Fig. 3B). The most abundant *Tetracladium* OTUs (19 and 1088) both showed a strong preference for the roots. The mean relative abundance of OTU 19 was five times higher in the roots than in the bulk soil and it was over a thousand times higher in the case of OTU 1088 (Additional file 4 D). Based on these results we conclude that OTUs 19 and 1088 can be categorised as putative root colonising fungi and further analyses focussed on these two abundant and widely distributed OTUs. Other root associated OTUs (OTUs 813, 1055, and 5882) could only be found in high abundance at sporadic sampling locations and were present in low abundance or were absent at most sites (Fig. 2).

### Correlating the metadata to OTU relative abundance

Relative abundance of OTUs 19 and 1088 in the root compartment had a significant positive linear correlation with oilseed rape (OSR, *Brassica napus*) yield (OTU 19 − R^2^ = 0.078, *P* =  < 0.001, OTU 1088 − R^2^ = 0.067, *P* =  < 0.001). The highest relative abundance of OTUs 19 and 1088 in the roots was associated with a yield increase of up to 25% relative to samples with the lowest relative abundance. No such relationship was seen in the bulk soil or rhizosphere (Fig. 3C).

**Fig. 4:**
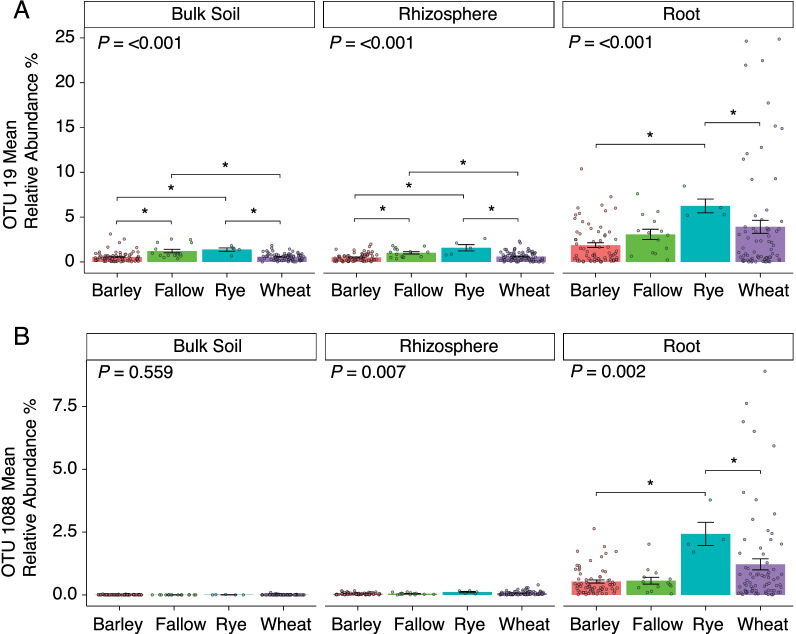
The mean relative abundance of **A** OTU 19 and **B** OTU 1088 in the four previous crop types in the bulk soil, the rhizosphere soil, and the roots. Stars indicate a significant difference between the previous crops. Error bars represent ± standard error of the mean

We further investigated the relationships between previous crop cultivated at the site, OSR variety and rotation and the relative abundance of OTUs 19 and 1088. We found that relative abundance of OTU 19 and 1088 had a significant correlation with previous cultivated crop (R^2^ = 0.021 *P* = 0.013 and R^2^ = 0.021, *P* = 0.012 respectively). In roots, relative abundance of both OTU 19 and 1088 was significantly higher (*P* < 0.001) when rye rather than barley or wheat was the proceeding crop (Fig. 4A). In the rhizosphere and soil relative abundance of OTU 19 showed the same patterns in relation to previous crops as for the roots, but in addition relative abundance following fallow was significantly higher than barley and lower than wheat (Fig. 4A, B, Additional file 5).

**Fig. 5:**
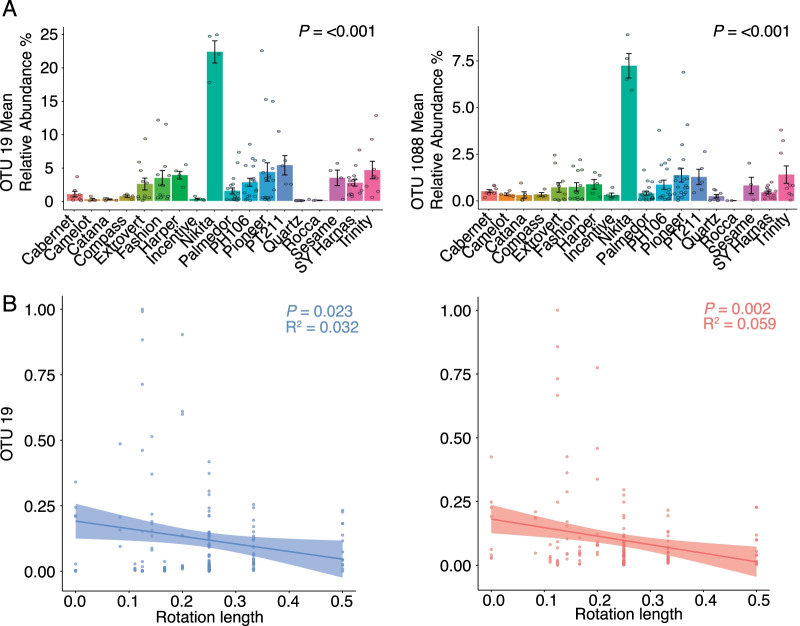
Relationships between *Tetracladium* spp. OTU mean relative abundance with variety and rotation. **A** The mean relative abundance of OTU 19 and 1088 in different OSR varieties in the roots. Error bars represent ± standard error of the mean. **B** Linear regression line fitted between OTU relative abundance and rotation length across the three compartments with significance values. OTU relative abundances and yield are normalised in a way to accommodate for fields that never had oilseed rape planted before. These virgin fields are represented as 0 while the shortest rotation length is represented by 0.5. The shaded region represents the 95% confidence limits for the estimated prediction

OSR variety had a significant effect on relative abundance of OTU 19 (R^2^ = 0.615, *P* =  < 0.001) and 1088 (R^2^ = 0.614, *P* =  < 0.001) in roots, with the two OTUs showing very similar distribution patterns. Seven varieties (Quartz, Rocca, Incentive, Compass, Catena, Camelot and Cabernet) had very low mean relative abundances of these OTUs, while Nikita had substantively higher relative abundance than the other varieties (Fig. 5A, B, Additional file 6). Rotation also had a significant linear correlation with the relative abundance of OTUs 19 and 1088 in the roots (R^2^ = 0.032, *P* = 0.023 for OTU 19, R^2^ = 0.059, *P* = 0.002 for OTU 1088) (Fig. 5B), which increased as time since previous OSR crop increased.

**Fig. 6:**
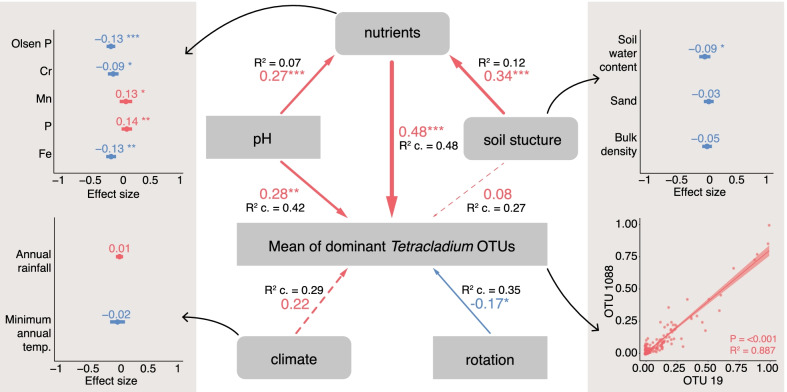
Drivers of the combined mean relative abundance of the two OTUs. Path diagram of the piecewise structural equation model (PSEM) showing direct and indirect effects with standard estimates R^2^ values for linear correlations and R^2^ conditional values for mixed effects linear correlations. The R^2^ marginal values for nutrients, pH, soil structure, climate, and rotation are: 0.35, 0.20, 0.03, 0.01, 0.03. Dotted lines indicate non-significance in the path model. Arrow sizes indicate effect size whereas arrow colours indicate a positive or a negative relationship (red—positive, blue—negative). The multivariate PSEM linking soil structure, pH, rotation, climate, and soil nutrients with mean relative abundance of OTUs 19 and 1088 was well supported by the data (Fisher's C = 36.86, *P* =  < 0.001, degrees of freedom = 10). *p < 0.05; **p < 0.01; ***p < 0.001. Standardised effect size is shown from the linear mixed effects models where soil compartment and sampled location were used as random effect. Model fit indicators for the LMMs are shown in Additional file 2. Rotation values are reciprocal

We found a strong correlation between the relative abundances of the root specific *Tetracladium* OTUs (R^2^ = 0.887, *P* =  < 0.001) so for further analyses we used the mean of the combined relative abundance of the two OTUs of each sample (Fig. 6). In the final PSEM, we correlated relative abundance of the combined OTUs to soil structure (soil moisture content, bulk density, and sand content), significant nutrients (Olsen P, iron, chromium, phosphorus, and manganese), pH, rotation, and climate (annual rainfall and minimum annual temperature) using sampled field as a random variable and used simple linear regression to correlate nutrients to soil structure and then pH to nutrients. Nutrient variables were chosen based on the results of the correlation matrix and the assessment of the model fit of the initial linear mixed-effect model modM (Additional file 1).

As a result, the PSEM showed the strongest significant direct positive effect of nutrients (R^2^ conditional = 0.48, standard estimate = 0.48, *P* = 0.001) followed by pH (R^2^ conditional = 0.07, standard estimate = 0.27, *P* = 0.002) and rotation (R^2^ conditional = 0.35, standard estimate = 0.17, *P* = 0.043) on the mean combined relative abundance of OTUs 19 and 1088 (Fig. 6). The effect of climate and soil structure was not significant in the path analyses. Soil structure and pH had significant positive correlations with nutrients (R^2^ = 0.12, standard estimate = 0.34, *P* =  < 0.001 and R^2^ conditional = 0.07, standard estimate = 0.27, *P* = 0.005) thus indirectly affecting OTU relative abundance. To expand the composite variables of the PSEM, the mixed-effect linear models showed significant correlation between the combined mean relative abundance of OTUs 19 and 1088 (Fig. 6) with Olsen P (*P* =  < 0.001), phosphorus (*P* = 0.003) and iron (*P* = 0.008). We also found a significant negative correlation between the combined mean relative abundance of the OTUs and soil water content (*P* = 0.045), while none of the climate variables were significant (Fig. 6).

## Discussion

Here we present the first systematic study of the landscape scale diversity and distribution of *Tetracladium* spp. within terrestrial systems and identify key factors controlling their occurrence as root endophytes. *Tetracladium* spp. were widely distributed, occurring in soil, rhizosphere, and roots in the 37 sampled sites. A total of 12 *Tetracladium* spp. OTUs were detected, and a subset of OTUs was specifically enriched in oilseed rape roots, including OTUs 19 and 1088 defined at 97% as *T. maxilliforme* and *T. furcatum.* Eight of the OTUs belonged to clades for which only environment sequences, largely from terrestrial habitats, have been recovered. There was a significant relationship between relative abundance of *T. maxilliforme* and *T. furcatum* within oilseed rape roots and crop genotype, previous cultivated crop, and oilseed rape rotation period. Linear mixed effects modelling and piecewise structural equation modelling showed that the most important environmental drivers of the relative abundance of *Tetracladium* spp. within plant roots were pH and select nutrients, including total phosphorus, extractable (Olsen) phosphorus, and iron.

### Diversity and distribution

*Tetracladium* is generally considered to be a freshwater fungi. However, members of the genus have also been detected as root endophytes of terrestrial plants. In the sampled fields there was lower species richness in the roots than in the soil and rhizosphere which is a common feature of endophytes, and suggests selective recruitment into the mycobiome [64]. Based on ITS sequences, the two most abundant OTUs (OTU 19 and 1088) clustered with *T. maxilliforme* and *T. furcatum* respectively and showed a strong root preference (Fig. 3B). *T. maxilliforme* and *T. furcatum* have been found several times in agriculture including the roots of crop plants [22, 25], though they are both traditionally regarded as aquatic organisms [14, 65–69]. OTU 813 clustered with the terrestrial *T. ellipsoideum* and it showed a strong root preference (Fig. 3B). Excluding OTUs 19, 1088, and 813, the closest GenBank sequence matches for all OTUs were from terrestrial habitats. Compartment preference did not have a correlation with taxonomic position as root-preferring OTUs were found across the main *Tetracladium* spp. clades. This suggests that the different species in the genus may not have one and the same defined lifestyle. Overall, our findings suggest that there is considerable diversity within terrestrial *Tetracladium* and that while some taxa may inhabit both aquatic and terrestrial habitats, others may inhabit terrestrial systems, with some occurring as plant endophytes.

Root endophytes have been shown to increase host resistance to environmental factors such as drought, heat and saline stress [70, 71], and can also increase plant health by inducing increased resistance via priming of the natural immune system to pathogens [72] or through competition with pathogens for nutrients and spatial niche exclusion [73]. Plant growth and yield can also be increased by endophytes via direct nutrient transfer from fungus to plant [74]. Furthermore, root colonising endophytes may shape plant community diversity and distribution [75].

Although *Tetracladium* species have previously been found in roots [19, 22, 25, 26], little is known about the process(es) by which *Tetracladium* spp. colonise roots, or the significance of infection for plant health. Sati and Arya [47] found that inoculation with *T. nainitalense* had no significant effect on the growth of *Hibiscus esculentus* or *Solanum melongena* following inoculation with *T. nainitalense,* although there was no evidence that the inoculant colonised plant tissues*.* In our previous work [38] we found a positive correlation between *Tetracladium* OTU relative abundance and oilseed rape yield on a landscape scale and in the current study we build on this to show a 25% yield increase from the lowest to the highest OTU relative abundance with both OTUs 19 and 1088. There is a clear need to understand the root infection process by *Tetracladium* spp. and to quantify benefits for plant health under controlled conditions, so that the significance of *Tetracladium* spp. root endophytes and their potential to act as beneficial symbionts can be established.

### Drivers of relative abundance

The root associated OTUs homologous to *T. maxilliforme* and *T. furcatum* OTUs showed strong co-assembly patterns in oilseed rape roots at a landscape scale. These OTUs showed the same interactions with host genotype, crop management, and environmental factors. There is evidence of minimal competition between root colonising endophytes explaining their high diversity within a single host [76]. This enables similar strains or species of fungi to colonise the same plant in high abundance. The data presented here could simply indicate similar adaptation of the two species without any ecological interaction.

According to our fitted models, the main drivers of *Tetracladium* sp. relative abundance in oilseed rape roots were soil nutrient content, crop rotation and pH. Relative abundance had a positive correlation with soil phosphorus and a negative correlation with iron content and Olsen P. Phosphorus and iron availability may limit plant and microbial growth in soil. For example, dark septate endophytes, which like *Tetracladium* spp. belong to the Helotiales, and occur as root endophytes, have been found to have iron phosphate solubilisation properties [77]. Sati and Pant found phosphate solubilisation in *T. setigerum* isolated from riparian roots in agar and broth media [78]. Moreover, it was shown that mineral fertilisers increased the relative abundance of *Tetracladium* sp. indicating that mineral fertiliser treatment might promote this plant-fungal symbiosis [23, 79]. In our study, total phosphorus content of the soil had a positive relationship with *Tetracladium* spp. OTU relative abundance in roots. In contrast, extractable phosphorus or Olsen P had a negative relationship with *Tetracladium* spp. OTU relative abundance in roots. This could suggest that *Tetracladium* spp. infection is promoted when bioavailability of P is low, in the same way that plants favour colonisation by arbuscular mycorrhizal symbioses under conditions of low P availability [80].

In contrast to bacteria, fungal communities are favoured by low soil pH [81]. However, OTUs homologous to *T. maxilliforme* and *T. furcatum* showed increased relative abundance in roots as soil pH increased. Furthermore, pH was a key factor in determining fungal community structure in the soil in many cases where *Tetracladium* has been identified as a common genus [41, 82], however it was found to prefer neutral or slightly acidic soil in a long-term microplot experiment [82]. In addition to pH, soil redox potential (Eh) is an important driver of microbial community growth, diversity and composition [83, 84]. Relative abundance of OTUs homologous to *T. maxilliforme* and *T. furcatum* increased as soil moisture decreased, which was surprising considering their dual ecology as aquatic taxa. Low soil moisture combined with high pH leads to lower Eh in the soil and results in slower rates of decomposition [85, 86]. In addition, high soil moisture and high Eh result in increased reducing conditions that limit extractable P availability in the soil through the solubilisation of Fe oxides that bind to available P [87]. Putative root colonising *Tetracladium* OTUs showed higher relative abundance in low reducing conditions (low soil moisture and high pH) and had higher relative abundance under low P availability, however this data originates from bulk soil physicochemical measurements and therefore the results may differ when looking at the soil closely encapsulating the roots.

Finally, we found a strong correlation between OTU relative abundance and crop management practices. OTUs 19 and 1088 both had the highest relative abundances in all compartments when OSR was planted after rye and their relative abundance increased with OSR rotation length. Crop rotation is known to influence soil microbial community composition, and to influence the composition of plant associated microbiota including pathogens and symbionts [88, 89]. These changes may be attributed to a wide range of interactions. For endophytes, they may reflect plant species specificity, and the extent to which different crop species support proliferation of inoculum, either following recruitment in living root biomass or on organic material left in the field following harvest [90]. Additionally, differences in management practices across crop types, such as fertiliser, tillage and pesticide use could also impact the inoculum [80]. Enhanced colonisation following ryegrass relative to OSR could therefore indicate a preference of *Tetracladium* spp. for ryegrass as a host, or management practices associated with ryegrass.

## Conclusion

The ecological interactions of *Tetracladium* spp. are a currently unknown; however, there is overwhelming evidence that some taxa within this group which were traditionally considered to be aquatic hyphomycetes can also occur as endophytes in terrestrial ecosystems, with several clades known only from environmental DNA, and which may represent terrestrial species. We found a correlation between crop yield and *Tetracladium* abundance, indicating that these fungi are signatures of a beneficial plant mycobiome. There is also indication that crop management practices, pH and nutrient enrichment are the main drivers of root colonisation of *Tetracladium* spp. in terrestrial environments. Further research is needed to determine their role in the plant’s life, particularly their effects on plant health and nutrition, to establish their potential value for utilisation in sustainable agricultural practices.

## Supplementary Information

**Additional file 1.** Correlogram showing Pearson’s correlation of all metadata variables and OTU relative abundance. Boxes are coloured according to the R values blue indicating a negative, red indicating a positive relationship. Non-significant relations are shown with an x in the box.

**Additional file 2.** Model indicators for LMMs. A is a visual representation of the measured model fit indices for the nutrient models. The final model included in Figure 6 is modMr. B is the actual values corresponding to the indicators of the nutrient models. C is the model fit indicators of the soil structure and climate models from Figure 6.

**Additional file 3.** Sequencing efficacy of the samples for the *Tetracladium* sp. OTUs. Rarefaction curves showing *Tetracladium* sp. OTU richness across all samples in the bulk soil, the rhizosphere, and the roots.

**Additional file 4.** Metadata tables. A - Metadata table from the 25 farms. B – Bulk soil properties from each five reps of the 37 field sites. C - Tetracladium OTU relative abundances across samples. D – Mean Tetracladium OTU relative abundances across the three sampled compartments. E – Closest sequence matches in the UNITE database with DOIs.

**Additional file 5.** Dunn’s test results correcting for P values with Benjamini–Hochberg method. Comparing previous crop types across the different compartments in OTU 19 and OTU 1088.

**Additional file 6.** Dunn’s test results correcting for P values with Benjamini–Hochberg method. Comparing OTU 19 and OTU 1088 relative abundance in different oilseed rape varieties in the roots.

**Additional file 7.** Multiple sequence alignment of the twelve *Tetracladium* sp. OTUs and the reference sequences.

## Data Availability

All data generated or analysed during this study are included in this published article [and its supplementary information files] and in Hilton, S., Picot, E., Schreiter, S. et al. Identification of microbial signatures linked to oilseed rape yield decline at the landscape scale. Microbiome 9, 19 (2021). https://doi.org/10.1186/s40168-020-00972-0. The sequences used for this study are deposited in the NCBI GenBank database under accession numbers MN047188, MN047207, and ON782228-ON782237. The multiple sequence alignment is provided in Additional file 7.
