# Supplementary figures and images for: Landscape scale ecology of Tetracladium spp. fungal root endophytes
Source: Environ Microbiome. 2022 Jul 25;17:40. doi: 10.1186/s40793-022-00431-3 (PMC9310467; doi:10.1186/s40793-022-00431-3)

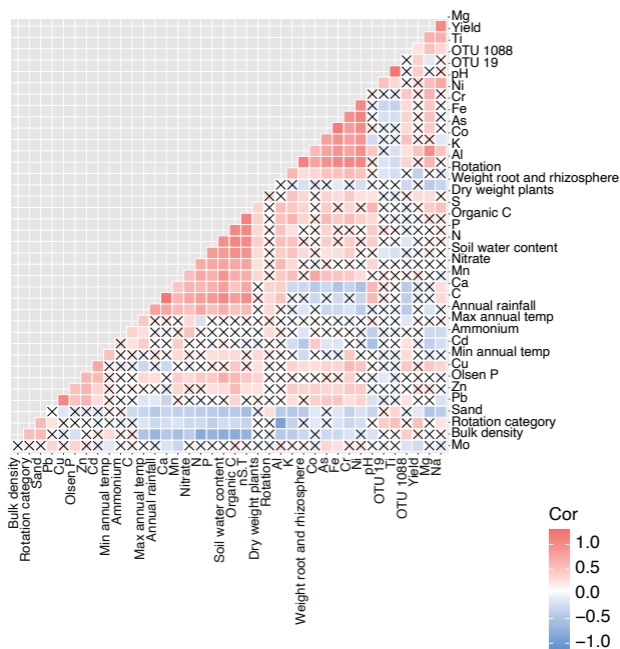

Supplement: Supplementary file 1 — Additional file 1. Correlogram showing Pearson’s correlation of all metadata variables and OTU relative abundance. Boxes are coloured according to the R values blue indicating a negative, red indicating a positive relationship. Non-significant relations are shown with an x in the box. [file 40793_2022_431_MOESM1_ESM.pdf]

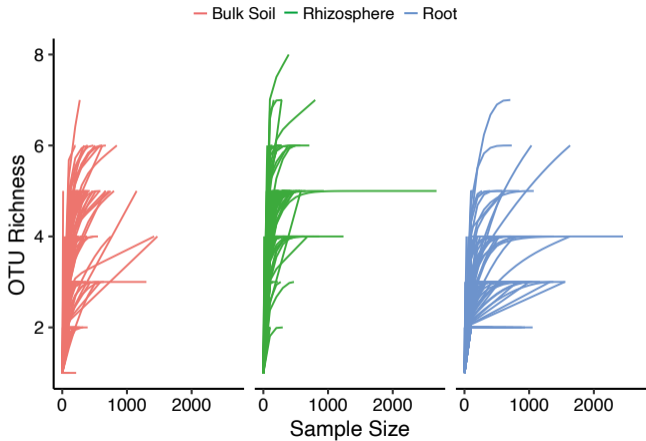

Supplement: Supplementary file 3 — Additional file 3. Sequencing efficacy of the samples for the Tetracladium sp. OTUs. Rarefaction curves showing Tetracladium sp. OTU richness across all samples in the bulk soil, the rhizosphere, and the roots. [file 40793_2022_431_MOESM3_ESM.pdf]
