# Supplementary material for: Landscape scale ecology of Tetracladium spp. fungal root endophytes
Source: Environ Microbiome. 2022 Jul 25;17:40. doi: 10.1186/s40793-022-00431-3 (PMC9310467; doi:10.1186/s40793-022-00431-3)
Supplement: Supplementary file 2 — Additional file 2. Model indicators for LMMs. A is a visual representation of the measured model fit indices for the nutrient models. The final model included in Figure 6 is modMr. B is the actual values corresponding to the indicators of the nutrient models. C is the model fit indicators of the soil structure and climate models from Figure 6. [file 40793_2022_431_MOESM2_ESM.pdf]

A

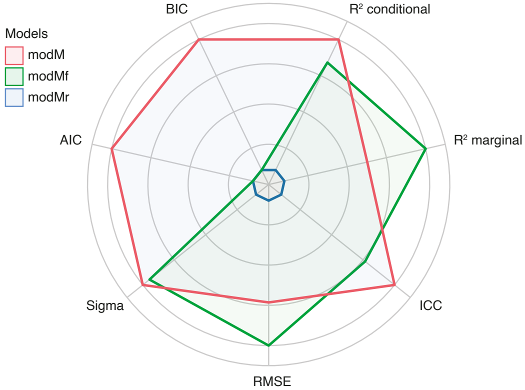

B

Name	R^2 conditional	R^2 marginal	ICC	RMSE	Sigma	AIC	BIC	Performance score
modMr	0.528	0.183	0.422	0.085	0.087	<0.001	<0.001	0.898
modMf	0.507	0.195	0.387	0.084	0.088	<0.001	<0.001	0.643
modM	0.409	0.167	0.290	0.088	0.090	<0.001	<0.001	0.000

C

modsoil	0.413	0.011	0.407	0.089	0.091
modclimate	0.407	0.002	0.406	0.089	0.091
