# Supplementary material for: Landscape scale ecology of Tetracladium spp. fungal root endophytes
Source: Environ Microbiome. 2022 Jul 25;17:40. doi: 10.1186/s40793-022-00431-3 (PMC9310467; doi:10.1186/s40793-022-00431-3)
Supplement: Supplementary file 7 — Additional file 7. Multiple sequence alignment of the twelve Tetracladium sp. OTUs and the reference sequences. [file 40793_2022_431_MOESM7_ESM.pdf]

```
>OTU_19
------------------------------------------------------------
------------------------------------------------------------
------------------------------------------------------------
------------------------------------------------------------
------------------------------------------------------------
------------------------------------------------------------
------------------------------------------------------------
------------------------------------------------------------
------------------------------------------------------------
------------------------------------------------------------
------------------------------------------------------------
------------------------------------------------------------
------------------------------------------------------------
------------------------------------------------------------
------------------------------------------------------------
------------------------------------------------------------
------------------------------------------------------------
------------------------------------------------------------
------------------------------------------------------------
------------------------------------------------------------
------------------------------------------------------------
------------------------------------------------------------
------------------------------------------------------------
------------------------------------------------------------
------------------------------------------------------------
------------------------------------------------------------
------------------------------------------------------------
------------------------------------------------------------
------------------------------------------------------------
------------------------------------------------------------
------------------------------------------------------------
------------------------------------------------------------
------------------------------------------------------------
-----------------------aacgcacattgcgccccttggtattccgaggggcatg
cctattcgagcgtcattatcacccctcaagc-ct-agcttggtgttgagacc-tgctgt-
caaggcagtctctaaaatcagtggcagt-gctgtcaggctctaagcgtagt-aaattcat
cgctatagacacctggtggccactcgccagaacccccccatttttt-aatgattgacctc
ggattaggtagggatacccgctgaacttaa------------------------------
------------------------------------------------------------
------------------------------------------------------------
------------------------------------------------------------
------------------------------------------------------------
------------------------------------------------------------
------------------------------------------------------------
------------------------------------------------------------
------------------------------------------------------------
------------------------------------------------------------
------------------------------------------------------------
------------------------------------------------------------
------------------------------------------------------------
------------------------------------------------------------
------------------------------------------------------------
------------------------------------------------------------
```


>OTU_168

-----------------------aacgcacattgcgccccctggtattccggggggcatg
cctattcgagcgtcattatcacccctcaagc-ct-agcttggtgttgaggcc-tgctgt-
caaggcagcctctaaaatcagtggcagt-gctgtcaggctctaagcgtagtaaaataaat
cgctatagcgtcctggtggacactcgtcagaaccccccattttt-aatgattgacctc
ggattaggtagggatacccgctgaacttaa------------------------------

>OTU_359

-----------------------aacgcacattgcgccccttggtattccgaggggcatg
cctattcgagcgtcattatcacccctcaagc-ct-agcttggtgttggggcc-tgccgt-
caaggcagcccttaaaatcagtggcagt-gctgtcaggctctaagcgtagtaa-acacgt
cgctatggagccctgg-gagcgctcgctatcaccccccacttttat-aatgattgacctc

ggattaggtagggatacccgctgaacttaa------------------------------

>OTU_813

-----------------------aacgcacattgcgccccttggtattccgaggggcatg
cctattcgagcgtcattatcacccctcaagctcc-ggcttggtgttgaggcc-tgctgt-
aaaggcaccctctaaaatcagtggcagt-gccgtcaggctctaagcgtagt-aatttcat
cgctatagac-ccgagcggaccctcgccagaaaccccccatttttt-aatgattgacctc
ggattaggtagggatacccgctgaacttaa------------------------------

>OTU_1055


```
------------------------------------------------------------
------------------------------------------------------------
------------------------------------------------------------
------------------------------------------------------------
------------------------------------------------------------
------------------------------------------------------------
------------------------------------------------------------
------------------------------------------------------------
------------------------------------------------------------
------------------------------------------------------------
------------------------------------------------------------
------------------------------------------------------------
-----------------------aacgcacattgcgccccttggtattccgaggggcatg
cctgttcgagcgtcattatcacccctcaagc-tc-tgcttggtgttggggcc-tgctgtg
ataggcagcccttaaaatcagtggcggt-gcctctcggctctaagcgtagtaattcctct
cgctatagtg-tccagtggttgctagccagaaccccccatttttt---catgattgacctc
ggatcaggtagggatacccgctgaacttaa------------------------------
------------------------------------------------------------
------------------------------------------------------------
------------------------------------------------------------
------------------------------------------------------------
------------------------------------------------------------
------------------------------------------------------------
------------------------------------------------------------
------------------------------------------------------------
------------------------------------------------------------
------------------------------------------------------------
------------------------------------------------------------
------------------------------------------------------------
------------------------------------------------------------
------------------------------------------------------------
------------------------------------------------------------
------------------------------------------------------------
------------------------------------------------------------
------------------------------------------------------------
------------------------------------------------------------
------------------------------------------------------------
------------------------------------------------------------
------------------------------------------------------------
------------------------------------------------------------
------------------------------------------------------------
------------------------------------------------------------
------------------------------------------------------------
------------------------------------------------------------
------------------------------------------------------------
------------------------------------------------------------
------------------------------------------------------------
------------------------------------------------------------
------------------------------------------------------------
------------------------------------------------------------
------------------------------------------------------------
------------------------------------------------------------
------------------------------------------------------------
------------------------------------------------------------
```


>OTU_1088

-----------------------aacgcaagttgcgccccttggtattccgaggggcatg
cctattcgagcgtcattatcacccctcaagc-ct-agcttggtgttgaggcc-tgctgt-
caaggcagcctctaaaatcagtggcagt-gctgtcaggctctaagcgtagtaaatctaat
cgctatagacacctggtggacactcgccagaacccccccatttttt-aatgattgacctc
ggattaggtagggatacccgctgagtttaa------------------------------

>OTU_3952

-----------------------aacgcacattgcgccccttggtattccgaggggcatg
cctgttcgagcgtcattatcacccctcaagc-tc-tgcttggtgttggggca-tgctgta
accggcaacccttaaaatcagtggcggt-gccatcgggctctaagcgtagtaacctctct
cgctacagtg-tccggtggttgctagccagaacccctaaatttt---tatgattgacctc
ggatcaggtagggatacccgctgaacttaa------------------------------

>OTU_4156

-----------------------aacgcacattgcgccccttggtattccgaggggcatg
cctattcgagcgtcattatcacccctcaagc-ct-agcttggtgttgagacc-tgctgt-
cagggcagtctctaaaatcagtggcagt-gctgtcaggctctaagcgtagt-aaattcat
cgctatagggtcctggtggacactcgccaaaacccccccatttttt-aatgattgacctc
ggattaggtagggatacccgctgagtttaa------------------------------

>OTU_5882

-----------------------aacgcacattgcgccccttggtattccgaggggcatg
cctattcgagcgtcattatcacccctcaagc-ct-agcttggtgttggggct-tgctgt-
aaaggcagcctctaaaatcagtggcagt-gctgtcaggctctaagcgtagt-aaattcat
cgctatagggtcctggtggatactcgtcaaaacccccc--attttt-aatgattgacctc
ggattaggtaggattacccgctgaacttaa------------------------------

>OTU_6656

-----------------------aacgcacattgcgccccttggtattccgaggggcatg
cctgttcgagcgtcattatcacccttcaagc-tt-tgcttggtgttggggca-tgccatc
acaggcagcccttaaaatcagtggcagtaaccattaggctctaagcgtagtaattcttct
cgctatagcg-tctggtggtcgcgcgccaaaacccccccatttt---catgattgacctc
ggatcaggtagggatacccgctgaacttaa------------------------------

>OTU_6663

-----------------------aacgcacattgcgccccttggtattccgaggggcatg
cctattcgagcgtcattatcacccctcaagc-ct-agcttggtgttgaggcc-tgctgt-
taaggcagcctctaaaatcagtggcagt-gctgtcaggctctaagcgtagt-aatttcat
cgctatagggtcctggtggatactcgccaaaacccccccatttttcaaatgattgacctc
ggattaggtagggatacccgctgaacttaa------------------------------

>OTU_10312

-----------------------aacgcacattgcgccccttggtattccggggggcatg
cctgttcgagcgtcattatcacccctcaagc-tc-agcttggtgttggggcc-tgctgta
aaaggcagcccttaaagtcagtggcggt-gctgtcaggctctaagcgtagtaacatatct
cgctatagggtcctggtggtgtcttgccaaaaccccacactttttt-aatgattgacctc
ggatcaggtagggatacccgctgaacttaa------------------------------

>KC180668.1 Tetracladium furcatum isolate GMU_LL_01_F4


```
------------------------------------------------------------
------------------------------------------------------------
------------------------------------------------------------
------------------------------------------------------------
------------------------------------------------------------
------------------------------------------------------------
------------------------------------------------------------
------------------------------------------------------------
------------------------------------------------------------
------------------------------------------------------------
------------------------------------------------------------
------------------------------------------------------------
------------------------------------------------------------
------------------------------------------------------------
------------------------------------------------------------
------------------------------------------------------------
------------------------------------------------------------
-------------------------------attaccgagttcatgccct---tacgggt
agatctcccaccctttgtata-ctatacct--ttgttgctttggcgggcc--gcctagct
a--------ctggcttcggctggtatgtgcccgccaga-ggacccaaaaccct-ga--at
tat--tagtgtcgtctgagtaaaata--tttaatatttaaaactttcaacaac-ggatct
cttggctctggcatcgatgaagaacgcagcgaaatgcgataagtaatgtgaattgcagaa
ttcagtgaatcatcgaatctttgaacgcacattgcgccccttggtattccgaggggcatg
cctattcgagcgtcattatcacccctcaagc-ct-agcttggtgttgaggcc-tgctgt-
caaggcagcctctaaaatcagtggcagt-gctgtcaggctctaagcgtagtaaatctaat
cgctatagacacctggtggacactcgccagaacccccccatttttt-aatgattgacctc
ggattaggtagggatacccgctgaacttaagcatatca----------------------
------------------------------------------------------------
------------------------------------------------------------
------------------------------------------------------------
------------------------------------------------------------
------------------------------------------------------------
------------------------------------------------------------
------------------------------------------------------------
------------------------------------------------------------
------------------------------------------------------------
------------------------------------------------------------
------------------------------------------------------------
------------------------------------------------------------
------------------------------------------------------------
------------------------------------------------------------
------------------------------------------------------------
------------------------------------------------------------
------------------------------------------------------------
------------------------------------------------------------
------------------------------------------------------------
------------------------------------------------------------
------------------------------------------------------------
------------------------------------------------------------
------------------------------------------------------------
------------------------------------------------------------
------------------------------------------------------------
------------------------------------------------------------
------------------------------------------------------------
```


>HQ623452.1 Tetracladium furcatum isolate N052

----ctcccaccctttgtata-ctatacct--ttgttgctttggcgggcc--gcctagct

```
a--------ctggcttcggctggtatgtgcccgccaga-ggacccaaaaccct-ga--at
tat--tagtgtcgtctgagtaaaata--tttaatatttaaaactttcaacaac-ggatct
cttggctctggcatcgatgaagaacgcagcgaaatgcgataagtaatgtgaattgcagaa
ttcagtgaatcatcgaatctttgaacgcacattgcgccccttggtattccgaggggcatg
cctattcgagcgtcattatcacccctcaagc-ct-agcttggtgttgaggcc-tgctgt-
caaggcagcctctaaaatcagtggcagt-gctgtcaggctctaagcgtagtaaatctaat
cgctatagacacctggtggacactcgccagaacccccccatttttt-aatgattgacctc
ggattaggtagggatacccgctgaacttaagcatatc-----------------------
------------------------------------------------------------
------------------------------------------------------------
------------------------------------------------------------
------------------------------------------------------------
------------------------------------------------------------
------------------------------------------------------------
------------------------------------------------------------
------------------------------------------------------------
------------------------------------------------------------
------------------------------------------------------------
------------------------------------------------------------
------------------------------------------------------------
------------------------------------------------------------
------------------------------------------------------------
------------------------------------------------------------
------------------------------------------------------------
------------------------------------------------------------
------------------------------------------------------------
------------------------------------------------------------
------------------------------------------------------------
------------------------------------------------------------
------------------------------------------------------------
------------------------------------------------------------
------------------------------------------------------------
------------------------------------------------------------
------------------------------------------------------------
------------------------------------------------------------
------------------------------------------------------------
------------------------------------------------------------
------------------------------------------------------------
------------------------------------------------------------
------------------------------------------------------------
------------------------------------------------------------
------------------------------------------------------------
------------------------------------------------------------
------------------------------------------------------------
------------------------------------------------------------
------------------------------------------------------------
------------------------------------------------------------
------------------------------------------------------------
------------------------------------------------------------
------------------------------------------------------------
------------------------------------------------------------
------------------------------------------------------------
------------------------------------------------------------
------------------------------------------------------------
```


>AF411027.1 Tetracladium maxilliforme strain F-14286

---------------------gcggaagatcattaccgagttcatgcccctt---cggggt
agatctcccaccctttgtata-ctatacct--ttgttgctttggcgggcc--gcctagct
a--------ctggcttcggctggtaagtgcccgccaga-gaacccaaaaccct-ga--at
tat--tagtgncgtctgagtaaaata--tttaatatttaaaactttcaacaac-ggatct
cttggctctggcatcgatgaagaacgcagcgaaatgcgataagtaatgtgaattgcagaa
ttcagtgaatcatcgaatctttgaacgcacattgcgccccttggtattccgaggggcatg
cctattcgagcgtcattatcacccctcaagc-ct-agcttggtgttgagacc-tgctgt-
caaggcagtctctaaaatcagtggcagt-gctgtcaggctctaagcgtant-aaattcat
cgctatagacacctggnggacactcgccagaa-ccccccatttttt-aatgattgacctc
ggattaggtagggatacccgctgaacttaa------------------------------

>KU519119.1 Tetracladium maxilliforme strain CCM F-529

---t
agatctcccaccctttgtata-ctatacct--ttgttgctttggcgggcc--gcctagct
a--------ctggcttcggctggtaagtgcccgccaga-gaacccaaaaccct-ga--at
tat--tagtgtcgtctgagtaaaata--tttaatatttaaaactttcaacaac-ggatct
cttggctctggcatcgatgaagaacgcagcgaaatgcgataagtaatgtgaattgcagaa
ttcagtgaatcatcgaatctttgaacgcacattgcgccccttggtattccgaggggcatg
cctattcgagcgtcattatcacccctcaagc-ct-agcttggtgttgagacc-tgctgt-
caaggcagtctctaaaatcagtggcagt-gctgtcaggctctaagcgtagt-aaattcat
cgctatagacacctggtggacactcgccagaa-ccccccatttttt-aatgattgacctc
ggattaggtagggatacccgctgaacttaagcatat------------------------

>AF411025.1 Tetracladium apiense strain F-23199

---------------------gcggaagatcattaccgagttcatgccct---tacgggt
agatctcccaccctttgtata-ccttacct--ttgttgctttggcgggcc--gcctagct
a--------ctggcttcggctggtangtgcccgccagagggacccaaaaccctgga--at
tat--tagtgtcgtctgagtaaaata--tttaatatttaaaactttcaacaacgggatct
cttggctctggcatcgatgaagaacgcagcgaaatgcgataagtaatgtgaattgcagaa

```
ttcagtgaatcatcgaatctttgaacgcacattgcgccccttggtattccgaggggcatg
cctattcgagcgtcattatcacccctcaagc-ct-agcttggtgttgaggcc-tgctgt-
caaggcagcctctaaaatcagtggcagt-gctgtcaggctctaagcgtagtaaacttcat
cgctatagacacctggtggacactcgccagaa-ccccccatttttt-aatgattgacctc
ggattaagtagggatacccgctgaacttaa------------------------------
------------------------------------------------------------
------------------------------------------------------------
------------------------------------------------------------
------------------------------------------------------------
------------------------------------------------------------
------------------------------------------------------------
------------------------------------------------------------
------------------------------------------------------------
------------------------------------------------------------
------------------------------------------------------------
------------------------------------------------------------
------------------------------------------------------------
------------------------------------------------------------
------------------------------------------------------------
------------------------------------------------------------
------------------------------------------------------------
------------------------------------------------------------
------------------------------------------------------------
------------------------------------------------------------
------------------------------------------------------------
------------------------------------------------------------
------------------------------------------------------------
------------------------------------------------------------
------------------------------------------------------------
------------------------------------------------------------
------------------------------------------------------------
------------------------------------------------------------
------------------------------------------------------------
------------------------------------------------------------
------------------------------------------------------------
------------------------------------------------------------
------------------------------------------------------------
------------------------------------------------------------
------------------------------------------------------------
------------------------------------------------------------
------------------------------------------------------------
------------------------------------------------------------
------------------------------------------------------------
------------------------------------------------------------
------------------------------------------------------------
------------------------------------------------------------
------------------------------------------------------------
------------------------------------------------------------
------------------------------------------------------------
------------------------------------------------------------
------------------------------------------------------------
------------------------------------------------------------
------------------------------------------------------------
------------------------------------------------------------
```

>FJ000369.1 Tetracladium apiense strain CCM F-23399

--------------------gcggaaggatcattaccgagttcatgcccctt-aaacgggt
agatctcccaccctttgtata-ccttacct--ttgttgctttggcgggcc--gcctagct
a--------ctggcttcggctggtaagtgcccgccaga-ggacccaaaaccct-ga--at
tat--tcgtgtcgtctgagtaaaatatttttaatatttaaaactttcaacaac-ggatct
cttggctctggcatcgatgaagaacgcagcgaaatgcgataagtaatgtgaattgcagaa
ttcagtgaatcatcgaatctttgaacgcacattgcgccccttggtattccgaggggcatg
cctattcgagcgtcattatcacccctcaagc-tc-agcttggtgttgaggcc-tgctgt-
gaaggcagcctctaaaatcagtggcagt-gctgtcaggctctaagcgtagtaaacttcat
cgctatagacaccggatggacactcgccagaa-ccccccatctttt-aatgattgacctc
ggattaggtagggatacccgctgaacttaagcat--------------------------

>AF411023.1 Tetracladium marchalianum strain F-312

---------------------gcggaagatcattaccgagttcatgccctataaacgggt
agatctcccaccctttgtatacctttacct--ttgttgctttggcgggcc--gcctagct
a--------ctggcttcngctggtaagtgcccgccaga-ggacccaaaaccct-ga--at
tat--tagtgtcgncngagtaaaata--tttaatatttaaaactttcaacaac-ggatct
cttggctctggcatcgatgaagaacgcagcgaaatgcgataagtaatgtgaattgcanaa
ttcagtgaatcatcgaatctttgaacgcacattgcgccccttggtattccgaggggcatg
cctattcgagcgtcattatcacccctcaagc-tc-agcttggtgttgaggcc-tgctgt-
caaggcagcctctaaaagcagtggcagt-gctgtcaggctctaagcgtagtaatctctct
cgctacagacacctgatggacactcgccagaa-ccccccatctttt-aatgattgacctc
ggattaggtagggatacccgctgaacttaa------------------------------

>KX858639.1 Tetracladium marchalianum isolate TEMA 1-203

------------------------aaggatcattaccgagttcatgccctataaacgggt
agatctcccaccctttgtatacctttacct--ttgttgctttggcgggcc--gcctagct
a--------ctggcttcggctggtaagtgcccgccaga-ggacccaaaaccct-ga--at
tat--tagtgtcgtctgagtaaaata--tttaatatttaaaactttcaacaac-ggatct
cttggctctggcatcgatgaagaacgcagcgaaatgcgataagtaatgtgaattgcagaa
ttcagtgaatcatcgaatctttgaacgcacattgcgccccttggtattccgaggggcatg
cctattcgagcgtcattatcacccctcaagc-tc-agcttggtgttgaggcc-tgctgt-
caaggcagcctctaaaagcagtggcagt-gctgtcaggctctaagcgtagtaatctctct

cgctacagacacctgatggacactcgccagaacccccccatctttt-aatga-tgacctc
gga---

>MH857378.1 Tetracladium setigerum strain CBS 422.54

-gtttccgtaggtg-aacctgcggaaggatcattaccgagttcatgccctt-aaacgggt
agatctcccacccttcgtata-ccttacct--ttgttgctttggcgggcc--gcctagct
a--------ctggcttcggctggtaagtgcccgccaga-ggacccaaaaccct-ga--at
tat--tcgtgtcgtctgagtaaaatatttttaatatttaaaactttcaacaac-ggatct
cttggctctggcatcgatgaagaacgcagcgaaatgcgataagtaatgtgaattgcagaa
ttcagtgaatcatcgaatctttgaacgcacattgcgccccttggtattccgaggggcatg
cctattcgagcgtcattatcacccctcaagc-tc-agcttggtgttgaggcc-tgctgt-
gaaggcagcctctaaaatcagtggcagt-gctgtcaggctctaagcgtagtaaacttcat
cgctataga---

>KU519120.1 Tetracladium setigerum strain CCM F-20987

--------------------------ggatcattaccgagttcatgccct---tacgggt
agatctcccaccctttgtata-ctatacct--ttgttgctttggcgggcc--gcctagct
a--------ctggcttcggctggtaagtgcccgccaga-ggacccaaaaccct-ga--at
tat--tagtgtcgtctgagtaaaata--tttaatatttaaaactttcaacaac-ggatct
cttggctctggcatcgatgaagaacgcagcgaaatgcgataagtaatgtgaattgcagaa
ttcagtgaatcatcgaatctttgaacgcacattgcgccccttggtattccgaggggcatg
cctattcgagcgtcattatcacccctcaagc-ct-agcttggtgttgaggcc-tgctgt-
caaggcagcctctaaaatcagtggcagt-gctgtcaggctctaagcgtagtaaaattcat
cgctatagacacctggtggacactcgccagaaccccccatttttt-aatgattgacctc
ggattaggtagggatacccgctgaacttaagcatatcaataa------------------

>JX029133.1 Tetracladium globosum isolate 25

-------------tttgtata-ctatacct--ttgttgctttggcgggcc--gcctagct
a--------ctggcttcggctggtaagtgcccgccaga-ggacccaaaaccct-ga--a-
tat--tagtgtcgtctgagtaaaat---tttaatatttaaaactttcaacaac-ggatct
cttggctctggcatcgatgaagaacgcagcgaaatgcgataagtaatgtgaattgcagaa
ttcagtgaatcatcgaatctttgaacgcacattgcgccccttggtattccgaggggcatg
cctattcgagcgtcattatcacccctcaagc-ttcggcttggtgttgaggcc-tgctgt-
aaaggcaccctctaaaatcagtggcagt-gctgtcaggctctaagcgtagt-aatttcat
cgctatagggtcctggtggatactcgtcaaaacccccccatttttt-aatgattgacctc
ggattaggtagggatacccgctgaacttaagcatatcaataagcgg--------------

>JX029118.1 Tetracladium globosum isolate 24

-----tcccaccctttgtata-ccatacct--ttgttgctttggcgggcc--gcctagct
a--------ctggcttcggctggtaagtgcccgccaga-ggacccaaaaccct-ga--a-
tat--tagtgtcgtctgagtaaaat---tttaatatttaaaactttcaacaac-ggatct
cttggctctggcatcgatgaagaacgcagcgaaatgcgataagtaatgtgaattgcagaa
ttcagtgaatcatcgaatctttgaacgcacattgcgccccttggtattccgaggggcatg
cctattcgagcgtcattatcacccctcaagc-ttcggcttggtgttgaggcc-tgctgt-
aaaggcaccctctaaaatcagtggcagt-gctgtcaggctctaagcgtagt-aatttcat
cgctatagggtcctggtggatactcgtcaaaacccccccatttttt-aatgattgacctc
ggattaggtagggatacccgctgaacttaagcatatcaataagcgg--------------

>JX029129.1 Tetracladium psychrophilum isolate 376

--------------gtgtata-ccttacct--ttgttgctttggcgggcc--gcctagct
a--------ctggcttcggctggtaagtgcccgccaga-gaaccccaaaccca-aaccat
tat--cagtatcgtctgagtacaaat--tttaatatttaaaactttcaacaac-ggatct
cttggctctggcatcgatgaagaacgcagcgaaatgcgataagtaatgtgaattgcagaa
ttcagtgaatcatcgaatctttgaacgcacattgcgccccttggtattccgaggggcatg
cctattcgagcgtcattatcacccctcaagctcc-ggcttggtgttgaggcc-tgccgt-
cccggcaccctctaaaatcagtggcagt-gccctcaggctctaagcgtagtaa--tttat
cgctacagggtcccgtgggatgctcgccagaacccccca-ttttt-aatgattgacctc
ggattaggtagggatacccgctgaacttaagcatatcaataagcggaggaa---------

>JX029119.1 Tetracladium psychrophilum isolate 380

-----------cctgtgtata-ccttacct--ttgttgctttggcgggcc--gcctagct
a--------ctggcttcggctggtaagtgcccgccaga-gaaccccaaaccca-aaccat
tat--cagtatcgtctgagtaaaaaa--tttaatatttaaaactttcaacaac-ggatct
cttggctctggcatcgatgaagaacgcagcgaaatgcgataagtaatgtgaattgcagaa
ttcagtgaatcatcgaatctttgaacgcacattgcgccccttggtattccgaggggcatg
cctattcgagcgtcattatcacccctcaagctcc-ggcttggtgttgaggcc-tgccgt-
cccggcaccctctaaaatcagtggcagt-gccctcaggctctaagcgtagtaa--tttat
cgctatagggtcccgtgggatgctcgccagaacccccca--ttttt-aatgattgacctc
ggattaggtagggatacccgctgaacttaagcatatcaataagcggagg-----------

>JX029124.1 Tetracladium ellipsoideum isolate 21

--cttccgtaggtg-aacctgcggaaggatcattaccgagttcatgccct---cacgggt
agacctcccaccctttgtata-ccttacct-gtcgttgctctggcgggtg----------
-----------------------ctcgcgcccgccgga-caacttcaaaccca-aa--cc
cattatagtgttgtctgagaaaaaag--tttaatatttaaaactttcaacaac-ggatct
cttggctctggcatcgatgaagaacgcagcgaaatgcgataagtaatgtgaattgcagaa
ttcagtgaatcatcgaatctttgaacgcacattgcgccccttggtattccgaggggcatg
cctattcgagcgtcattatcacccctcaagctcc-ggcttggtgttgaggcc-tgctgt-
aaaggcaccctctaaaatcagtggcagt-gccgtcaggctctaagcgtagt-aatttcat
cgctatagat-ccgagcggaccctcgccagaaaccccccatttttt-aatgattgacctc
ggattaggtagggatacccgctgaacttaagcatatcaataagcggaggaa---------


```
------------------------------------------------------------
------------------------------------------------------------
------------------------------------------------------------
------------------------------------------------------------
------------------------------------------------------------
------------------------------------------------------------
------------------------------------------------------------
------------------------------------------------------------
------------------------------------------------------------
------------------------------------------------------------
------------------------------------------------------------
------------------------------------------------------------
------------------------------------------------------------
------------------------------------------------------------
------------------------------------------------------------
------------------------------------------------------------
------------------------------------------------------------
------------------------------------------------------------
------------------------------------------------------------
------------------------------------------------------------
------------------------------------------------------------
------------------------------------------------------------
------------------------------------------------------------
------------------------------------------------------------
------------------------------------------------------------
------------------------------------------------------------
--------------------------
>JX029113.1 Tetracladium ellipsoideum isolate 30m
------------------------------------------------------------
------------------------------------------------------------
------------------------------------------------------------
------------------------------------------------------------
------------------------------------------------------------
------------------------------------------------------------
------------------------------------------------------------
------------------------------------------------------------
------------------------------------------------------------
------------------------------------------------------------
------------------------------------------------------------
------------------------------------------------------------
------------------------------------------------------------
------------------------------------------------------------
------------------------------------------------------------
------------------------------------------------------------
------------------------------------------------------------
------------------------------------------------------------
------------------------------------------------------------
------------------------------------------------------------
------------------------------------------------------------
------------------------------------------------------------
------------------------------------------------------------
------------------------------------------------------------
------------------------------------------------------------
------------------------------------------------------------
```

```
------------------------------------------------------------
------------------------------------------------------------
--cttccgtaggggtaacctgcggaaggatcattaccgagttcatgccct---cacgggt
agacctcccaccctttgtata-ccttacct-cttgttgctccggtgggct-------tcg
a--------ccggcctcggctgggaagcgcccgccgga-cagc----atacca-aa--cc
cattatagtgtcgtctgagaaaaaag--tttaatatttaaaactttcaacaac-ggatct
cttggctctggcatcgatgaagaacgcagcgaaatgcgataagtaatgtgaattgcagaa
ttcagtgaatcatcgaatctttgaacgcacattgcgccccttggtattccgaggggcatg
cctattcgagcgtcattatcacccctcaagctcc-ggcttggtgttgaggcc-tgctgt-
aaaggcatcctctaaaatcagtggcagt-gccgtcaggctctaagcgtagt-aatttcgt
cgctatagat-cctggcggaccctcgccagaacccccccatttttt-aatgattgacctc
ggattaggtagggatacccgctgaacttaagcatatcaatagcccggaggaaagg-----
------------------------------------------------------------
------------------------------------------------------------
------------------------------------------------------------
------------------------------------------------------------
------------------------------------------------------------
------------------------------------------------------------
------------------------------------------------------------
------------------------------------------------------------
------------------------------------------------------------
------------------------------------------------------------
------------------------------------------------------------
------------------------------------------------------------
------------------------------------------------------------
------------------------------------------------------------
------------------------------------------------------------
------------------------------------------------------------
------------------------------------------------------------
------------------------------------------------------------
------------------------------------------------------------
------------------------------------------------------------
------------------------------------------------------------
------------------------------------------------------------
------------------------------------------------------------
------------------------------------------------------------
------------------------------------------------------------
------------------------------------------------------------
------------------------------------------------------------
------------------------------------------------------------
------------------------------------------------------------
------------------------------------------------------------
------------------------------------------------------------
------------------------------------------------------------
------------------------------------------------------------
------------------------------------------------------------
------------------------------------------------------------
------------------------------------------------------------
------------------------------------------------------------
------------------------------------------------------------
------------------------------------------------------------
------------------------------------------------------------
------------------------------------------------------------
------------------------------------------------------------
------------------------------------------------------------
```


>GQ411301.1 Tetracladium breve isolate UMB-046.01

------------------------aaggatcattaccgagttcatgccctg-aaaagggt
agatctcccaccctttgtata-ccttacct--ttgttgctttggcgggcc--gcctagct
a--------ctggcttcggctggtaagtgcccgccaga-ggacccaaaaccct-ga--at
tat--tagtgtcgtctgagtaaaata--tttaatatttaaaactttcaacaac-ggatct
cttggctctggcatcgatgaagaacgcagcgaaatgcgataagtaatgtgaattgcagaa
ttcagtgaatcatcgaatctttgaacgcacattgcgccccttggtattccgaggggcatg
cctattcgagcgtcattatcacccctcaagc-ttcagcttggtgttgaggcc-tgctgt-
caaggcagcctctaaaatcagtggcagt-gctgtcaggctctaagcgtagtaaaattcat
cgctatagacacctggtggacactcgccagaa--cccccaattttt-aatgattgacctc
ggattaggtagggatacccgctgaacttaa------------------------------

>MN864185.1 Tetracladium breve voucher PDD:110838


```
------------------------------------------------------------
------------------------------------------------------------
------------------------------------------------------------
------------------------------------------------------------
------------------------------------------------------------
------------------------------------------------------------
------------------------------------------------------------
------------------------------------------------------------
------------------------------------------------------------
------------------------------------------------------------
------------------------------------------------------------
------------------------------------------------------------
------------------------------------------------------------
------------------------------------------------------------
------------------------------------------------------------
------------------------------------------------------------
------------------------------------------------------------
---------------------------------------aggaagtaaaagtcgtaacaa
ggtttccgtaggtg-aacctgcggaaggatcattaccgagttcatgccctt-aaacgggt
agatctcccaccctttgtata-ccttacct--ttgttgctttggcgggcc--gcctagct
a--------ctggcttcggctggtaagtgcccgccaga-ggacccaaaaccct-ga--ag
tat--tagtgtcgtctgagtaaaata--tttaatatttaaaactttcaacaac-ggatct
cttggctctggcatcgatgaagaacgcagcgaaatgcgataagtaatgtgaattgcagaa
ttcagtgaatcatcgaatctttgaacgcacattgcgccccttggtattccgaggggcatg
cctattcgagcgtcattatcacccctcaagcttt-agcttggtgttgaggcc-tgctgt-
caaggcagcctctaaaatcagtggcagt-gctgtcaggctctaagcgtagtaaaattcat
cgctatagacaccggatggacactcgccagaaaccccccatctttt-aatggttgacctc
ggattaggtagggatacccgctgaacttaagcata-------------------------
------------------------------------------------------------
------------------------------------------------------------
------------------------------------------------------------
------------------------------------------------------------
------------------------------------------------------------
------------------------------------------------------------
------------------------------------------------------------
------------------------------------------------------------
------------------------------------------------------------
------------------------------------------------------------
------------------------------------------------------------
------------------------------------------------------------
------------------------------------------------------------
------------------------------------------------------------
------------------------------------------------------------
------------------------------------------------------------
------------------------------------------------------------
------------------------------------------------------------
------------------------------------------------------------
------------------------------------------------------------
------------------------------------------------------------
------------------------------------------------------------
------------------------------------------------------------
------------------------------------------------------------
------------------------------------------------------------
------------------------------------------------------------
```


>FJ000372.1 Tetracladium palmatum strain CCM F-10001

---------------------cggaaggatcattaccgagttcatgccct---tacgggt

agatctcccaccctttgtata-ctatacct--ttgttgctttggcgggcc--gcctagct
a--------ctggcttcggctggtaagtgcccgccaga-ggacccaaaaccct-ga--at
tat--tagtgtcgtctgagtaaaata-ttttaatatttaaaactttcaacaac-ggatct
cttggctctggcatcgatgaagaacgcagcgaaatgcgataagtaatgtgaattgcagaa
ttcagtgaatcatcgaatctttgaacgcacattgcgccccttggtattccgaggggcatg
cctattcgagcgtcattatcacccctcaagc-tt-agcttggtgttgaggtc-tgctgt-
caaggcagcctctaaaatcagtggcagt-gctgtcaggctctaagcgtagtaaaattcat
cgctatagacacctggtggacactcgccagaacccccccatttttt-aatgattgacctc
ggattaggtagggatacccgctgaacttaagcat--------------------------

>EU883424.1 Tetracladium palmatum strain CCM F-10001
aactgcgaatggctcattaaatcagttatcgtttatttgatagtaccttactacttggat
aaccgtggtaattctagagctaatacatgctaaaaaccccgacttttggaggggtgtatt
tattagataaaaaaccaatgcccttcggggctccttggtgattcataataacttaacgaa
tcgcatggccttgtgccggcgatggttcattcaaatttctgccctatcaactttcgatgg
ttaggtcttggctaaccatggtttcaacgggtaacggggaattagggttctattccggag
agtgagcctgagaaacggctaacacatccaaggaaggcagcaggcgcgcaaattacccaa
tcccgacacggggaggtagtgacaataaatactgatccagggctcttttgggtcttggaa
ttggaatgagtacaatttaaatcccttaacgaggaacaattggagggcaagtctggtgcc
agcagccgcggtaattccagctccaatagcgtatattaaagttgttgcagttaaaaagct
cgtagttgaaccttgggtctggctggccggtccgcctcaccgcgtgtactggtccggccg
gacctttccttctggggaatcgcatgcccttcactgggtgtgtcgaggatccaggacttt
tactttgaaaaaattagagtgttcaaagcaggcctatgctcgaatacattagcatggaat
aatagaataggacgtgtggttctattttgttggtttctaggaccgccgtaatgattaata
gggatagtcgggggcatcagtattcaattgtcagaggtgaaattcttggatttattgaag
actaactactgcgaaagcatttgccaaggatgttttcattaatcagtgaacgaaagttag
gggatcgaagacgatcagataccgtcgtagtcttaaccataaactatgccgactagggat
cgggcgatgttactttttttgactcgctcggcaccttacgagaaatcaaagtctttgggtt
ctgggggagtatggtcgcaaggctgaaacttaaagaaattgacggaagggcaccaccag
gagtggagcctgcggcttaatttgactcaacacggggaaactcaccaggtccagacacaa
taaggattgacagattgagagctctttcttgattttgtgggtggtggtgcatggccgttc
ttagttggtggagtgatttgtctgcttaattgcgataacgaacgagactttgacttttaa
atagctaggctagctttggctggtcgctggcttcttagaaggactatttgctcaagcaaa
tggaagtgcgaagcaataacaggtctgtgatgcccttagatgttctgggccgcacgcgcg
ctacactgacagagccaacgagttcttccttagccgaaaggtttgggtaatcttgttaaa
ctctgtcgtgctggggatagagcattgcaattattgctcttcaacgaggaattcctagta
agcgcaagtcatcagcttgcgctgattacgtccctgccctttgtacacaccgcccgtcgc
tactaccgattgaatgattcagtgaggctttcggactggcccaggaagagtggcaacact
catctagggccggaaagttgtccaaacttggtcatttagaggaagtaaaagtcgtaacaa
ggtttccgtaggtg-aacctgcggaaggatcattaccgagttcatgccctataaacgggt
agatctcccaccctttgtatacctatacct--ttgttgctttggcgggcc--gcctagct
a--------ctggcttcggctggtaagtgcccgccaga-ggacccaaaccct-ga--at
tat--tagtgtcgtctgagtaaaatatttttaatatttaaaactttcaacaac-ggatct
cttggctctggcatcgatgaagaacgcagcgaaatgcgataagtaatgtgaattgcagaa
ttcagtgaatcatcgaatctttgaacgcacattgcgccccttggtattccgagggcatg
cctattcgagcgtcattatcacccctcaagc-ctcagcttggtgttgaggcc-tgctgt-
caaggcagcctctaaaatcagtggcagt-gctgtcaggctctaagcgtagtaaaattcat
cgctatagacacctggtggacactcgccagaaccccccatttttt-aatgattgacctc
ggattaggtagggatacccgctgaacttaagcatatcaataagcggaggaaaagaaacca
acagggattgcctcagtaacggcgagtgaagcggcaaaagctcaaatttgaaatctggct
cttttagggtccgag-ttgtaatttgtagaagat-gtttcgggtgtggctccggtttaag
ttctttggaatattacatcatagagggtgagaatcccgtatgtgaccggcagccttcgcc
tatgtgaaactctttcgacgagtcgagttgtttgggaatgcagctcaaaatgggaggtat
atttcttctaaagctaaatattggccagagaccgatagcgcacaagtagagtgatcgaaa
gatgaaaagcactttggaaagagagttaaacagtacgtgaaattgttgaaagggaagcgc
ttgcaaccagacttgcacgcagttgatcatccggtgttctcaccggggcactctgctgcg
ttcaggccagcatcggttttggtggttggataaaggccttgggaatgtggcttccttcgg
ggagtgttatagccctcggtgcaatgcagcctaccgggaccgaggaccgcgcttcggcta
ggatgctggcgtaatggttgtaagcgacccgtcttgaaacacggaccaaggagtctaaca

tctatgcgagtgtttgggtgttaaacccatacgcgtaatgaaagtgaacggaggtgagaa
cccttaagggtgcatcatcgaccggtcctgatgtcttcggatggatctgagtaagagcat
agctgttgggacccgaaagatggtgaactatgcgtgaatagggtgaagccagaggaaact
ctggtggaggctcgcagcggttctgacgtgcaaatcgatcgtcaaatttgcgcataggggg
cgaaagacttatcgaaccatctagtagctggttcctgccgaagtttccctcaggatagca
gtgttgaattcagttttatgaggtaaagcgaatgattagaggccttggggttgaaacaac
cttaacctattctcaaactttaaatatgtaagaagtccttgttacttaattgaacgtgga
cattcgaatgtaccaacactagtgggccatttttggtaagcagaactggcgatgcgggat
gaaccgaacgtgaagttaaggtgccggaatatacgctcatcagacaccacaaaggtgtt
agttcatctagacagcaggacggtggccatggaagtcggaatccgctaaggaatgtgtaa
caactcacctgccgaatgaactagccctgaaaatggatggcgcttaagcgtattacccat
acttcaccgccagggtagaaacgatgccctggcgagtaggcaggcgtggaggtcagtgac
gaagccttgggggtgaccccgggtagaacggcctctagtgcagatcttggtggtagtagc
aaatactcaaatgagaactttgaggactgaagtggggaaaggttccatgtgaacagcagt
tggacatgggttagtcgatcctaagcgatagggaaactccgttttaaatgtgcacttgtg
cactatcacgcgaaagggaagccggttaatattccggcacctggatttggattctccacg
gcaacgtaactgaacgcggagacgacggcgggggccctgggaagagttctcttttcttct
taacagcctatcaccctgaaatcggtttgtccggagctagggtttaatggttggtagagc
ctgacacctttgtcaggtccggtgcgctctcgacgtcccttgaaaatccgcgggaagaaa
tagctttcaagccaggtcgtactcataaccgcagcaggtctccaaggtgaacagcctcta
gttgatagaacaatgtagataagggaagtcggcaaaatagatccgtaacttcgggaaaag
gattggctctaagggttgggtacgttgggccttaggtggacgtctctggagcaggtcggc
actagcctcacggccggcgcctttcagcatcggggttctgacgcctttggcaggcttcgg
ccgtccggcgtacaattaacaaccaacttagaactggtacggacaaggggaatctgactg
tctaattaaaacatagcattgcgatggccagaaagtggtgttgacgcaatgtgatttctg
cccagtgctctgaatgtcaaagtgaagtaattcaaccaagcgcgggtaaacggcgggagt
aactatgactctcttaaggtagccaaatgcctcgtcatctaattagtgacgcgcatgaat
ggattaacgagattcccactgtccctatctactatctagcgaaaccacagccaagggaac
gggcttggcagaatcagcggggaaagaagaccctgttgagcttgactctagtttgacatt
gtgaaaagacataggggggtgtagaataggtgggagcgcaagcgccggtgaaataccacta
cccttatcgtttttttacttattcaataaagcggaactgggtgtcaaagcccaacttcta
gcattaaggtccttcgcgggctgatccgggttgaagacattgtcaggtggggagtttggc
tggggcggcacatctgttaaaccataacgcaggtgtcctaagggggactcatggagaaca
gaaatctccagtagaacaaaagggtaaaagtccccttgattttgattttcagtgtgaata
caaaccatgaaagtgtggcctatcgatcctttagtccctcgaaatttgaggctagaggtg
ccagaaaagttaccacagggataactggcttgtggcagccaagcgttcatagcgacgttg
ctttttgatccttcgatgtcggctcttcctatcataccgaagcagaattcggtaagcgtt
ggattgttcacccactaatagggaacgtgagctgggtttagaccgtcgtgagacaggtta
gttttaccctactgatgaccgtcaccgcaatggtaattcagcttagtacgagaggaaccg
ctgattcagataattggttttgcggc
>GU055746.1 Uncultured Tetracladium sp.


```
------------------------------------------------------------
------------------------------------------------------------
------------------------------------------------------------
------------------------------------------------------------
------------------------------------------------------------
------------------------------------------------------------
------------------------------------------------------------
------------------------------------------------------------
------------------------------------------------------------
------------------------------------------------------------
------------------------------------------------------------
------------------------------------------------------------
------------------------------------------------------------
------------------------------------------------------------
------------------------------------------------------------
------------------------aaggatcattaacgagttcatgcccc---tcggggt
agatctcccaccctttgtata-ctatacct--ttgttgctttggcgggcc--gcctagct
a--------ctggcttcggctggtaagtgcccgccaga-ggacccaaaccct-ga--a-
tat--tagtgtcgtctgagtaaaaag--tttaatatttaaaactttcaacaac-ggatct
cttggctctggcatcgatgaagaacgcagcgaaatgcgataagtaatgtgaattgcagaa
ttcagtgaatcatcgaatctttgaacgcacattgcgcccctggtattccgggggcatg
cctattcgagcgtcattatcacccctcaagc-ct-agcttggtgttgaggcc-tgctgt-
caaggcagcctctaaaatcagtggcagt-gctgtcaggctctaagcgtagtaaaataaat
cgctatagcgtcctggtggacactcgtcagaaccccccatttttt-aatgattgacctc
ggattaggtagggatacccgctgaacttaagcatatcaataagcggaggaaaagaaacca
acagggattgcctcagtaacggcgagtgaagcggcaaaagctcaaatttgaaatctggct
cttttagggtccgag-ttgtaatttgtagaagat-gtttcgggtgtggctccggtttaag
ttctttggaatattacatcatagagggtgagaatcccgtatgtgaccggcagcctccgcc
tatgtgaaactctttcgacgagtcgagttgtttgggaatgcagctcaaaatgggaggtat
atttcttctaaagctaaatattggccagagaccgatagcgcacaagtagagtgatcgaaa
gatgaaaagcactttggaaagagagttaaacagtacgtgaaattgttgaaagggaagcgc
ttgcaaccagacttgcacgcagttgatcatccggtgttctcaccggggcactctgctgcg
ttcaggccagcatcggttttggtggttggataaaggccttgggaatgtggcttccttcgg
ggagtgttatagccctcggtgcaatgcagcctacccggaccgaggaccgcgcttcggcta
ggatgctggcgtaatggttgtaagcgacc-------------------------------
------------------------------------------------------------
------------------------------------------------------------
------------------------------------------------------------
------------------------------------------------------------
------------------------------------------------------------
------------------------------------------------------------
------------------------------------------------------------
------------------------------------------------------------
------------------------------------------------------------
------------------------------------------------------------
------------------------------------------------------------
------------------------------------------------------------
------------------------------------------------------------
------------------------------------------------------------
------------------------------------------------------------
------------------------------------------------------------
------------------------------------------------------------
------------------------------------------------------------
------------------------------------------------------------
```


>MN660932.1 Uncultured Tetracladium sp.

-------------------------cgcacattgcgccccctggtattccggggggcatg
cctattcgagcgtcattatcacccctcaagc-ct-agcttggtgttgaggcc-tgctgt-
caaggcagcctctaaaatcagtggcagt-gctgtcaggctctaagcgtagtaaaataaat
cgctatagcgtcctggtggacactcgtcagaacccccccatttttt-aatgattgacctc
ggattaggtagggatacccgctgaacttaa------------------------------


```
------------------------------------------------------------
--------------------------
>LR876862.1 uncultured fungus
------------------------------------------------------------
------------------------------------------------------------
------------------------------------------------------------
------------------------------------------------------------
------------------------------------------------------------
------------------------------------------------------------
------------------------------------------------------------
------------------------------------------------------------
------------------------------------------------------------
------------------------------------------------------------
------------------------------------------------------------
------------------------------------------------------------
------------------------------------------------------------
------------------------------------------------------------
------------------------------------------------------------
------------------------------------------------------------
------------------------------------------------------------
------------------------------------------------------------
------------------------------------------------------------
------------------------------------------------------------
------------------------------------------------------------
------------------------------------------------------------
------------------------------------------------------------
------------------------------------------------------------
------------------------------------------------------------
------------------------------------------------------------
------------------------------------------------------------
------------------------------------------------------------
------------------------------------------------------------
------------------------------------------------------------
------------------------------------------------------------
------------------------------------------------------------
------------------------------------------------------------
----------------------gaacgcacattgcgccccttggtattccgaggggcatg
cctattcgagcgtcattatcacccctcaagc-ct-agcttggtgttggggcc-tgccgt-
caaggcagcccttaaaatcagtggcagt-gctgtcaggctctaagcgtagtaa-acacgt
cgctatggagccctgg-gagcgctcgctatcaccccccacttttat-aatgattgacctc
ggattaggtagggatacccgctgaacttaag-----------------------------
------------------------------------------------------------
------------------------------------------------------------
------------------------------------------------------------
------------------------------------------------------------
------------------------------------------------------------
------------------------------------------------------------
------------------------------------------------------------
------------------------------------------------------------
------------------------------------------------------------
------------------------------------------------------------
------------------------------------------------------------
------------------------------------------------------------
------------------------------------------------------------
```


>MK627349.1 Uncultured Tetracladium sp.


```
------------------------------------------------------------
------------------------------------------------------------
------------------------------------------------------------
------------------------------------------------------------
------------------------------------------------------------
------------------------------------------------------------
------------------------------------------------------------
------------------------------------------------------------
------------------------------------------------------------
------------------------------------------------------------
------------------------------------------------------------
------------------------------------------------------------
------------------------------------------------------------
------------------------------------------------------------
------------------------------------------------------------
------------------------------------------------------------
-----------------------------cgaaatgcgataagtaatgtgaattgcagaa
ttcagtgaatcatcgaatctttgaacgcacattgcgccccttggtattccgaggggcatg
cctattcgagcgtcattatcacccctcaagc-ct-agcttggtgttggggcc-tgccgt-
caaggcagcccttaaaatcagtggcggt-gctgtcaggctctaagcgtagtaa-acacgt
cgctatggagccctgg-gagcgctcgctatcaccccccacttttat-aatgattgacctc
ggattaggtagggatacccgctgaacttaagc----------------------------
------------------------------------------------------------
------------------------------------------------------------
------------------------------------------------------------
------------------------------------------------------------
------------------------------------------------------------
------------------------------------------------------------
------------------------------------------------------------
------------------------------------------------------------
------------------------------------------------------------
------------------------------------------------------------
------------------------------------------------------------
------------------------------------------------------------
------------------------------------------------------------
------------------------------------------------------------
------------------------------------------------------------
------------------------------------------------------------
------------------------------------------------------------
------------------------------------------------------------
------------------------------------------------------------
------------------------------------------------------------
------------------------------------------------------------
------------------------------------------------------------
------------------------------------------------------------
------------------------------------------------------------
------------------------------------------------------------
------------------------------------------------------------
------------------------------------------------------------
------------------------------------------------------------
------------------------------------------------------------
------------------------------------------------------------
------------------------------------------------------------
------------------------------------------------------------
```


>MK627297.1 Uncultured Tetracladium sp.

-----------------------------cgaaatgcgataagtaatgtgaattgcagaa
ttcagtgaatcatcgaatctttgaacgcacattgcgccccttggtattccgaggggcatg
cctattcgagcgtcattatcacccctcaagctcc-ggcttggtgttgaggcc-tgctgt-

aaaggcaccctctaaaatcagtggcagt-gccgtcaggctctaagcgtagt-aatttcat
cgctatagac-ccgagcggaccctcgccagaaaccccccatttttt-aatgattgacctc
ggattaggtagggatacccgctgaacttaagc----------------------------

>MF181805.1 Uncultured fungus

-----------------------aacgcacattgcgccccttggtattccgaggggcatg
cctattcgagcgtcattatcacccctcaagctcc-ggcttggtgttgaggcc-tgctgt-
aaaggcaccctctaaaatcagtggcagt-gccgtcaggctctaagcgtagt-aatttcat
cgctatagac-ccgagcggaccctcgccagaaaccccccatttttt-aatgattgacctc
ggattaggtagggatacccgctgaacttaa------------------------------

>MH451254.1 Uncultured fungus

-----------------------aacgcacattgcgccccttggtattccgaggggcatg
cctgttcgagcgtcattatcacccctcaagc-tt-tgcttggtgttggggcc-tgctgta
ataggcagcccttaaaatcagtggcggt-gcttcaaggctctaagcgtagtaattctcct
cgctatagtg-tctagtggttgccagccagaaccccccaatttt---catgattgacctc
ggatcaggtagggatacccgctgaacttaa------------------------------

>MW050202.1 Uncultured fungus

------------------------------gaaatgcgataagtaatgtgaattgcagaa
ttcagtgaatcatcgaatctttgaacgcacattgcgccccttggtattccgaggggcatg
cctgttcgagcgtcattatcacccctcaagc-tt-tgcttggtgttggggcc-tgctgta
ataggcagcccttaaaatcagtggcggt-gcttcaaggctctaagcgtagtaattctcct
cgctatagtg-tctagtggttaccagccagaaccccccaatttt---catgattgacctc
ggatcaggtagggatacccgctgaacttaa------------------------------

>KX193670.1 Uncultured fungus

------------------------------gaaatgcgataagtaatgtgaattgcagaa
ttcagtgaatcatcgaatctttgaacgcacattgcgccccttggtattccgaggggcatg
cctgttcgagcgtcattatcacccctcaagc-gt-ggcttggtattggggcc-tgctgta
actggcagcccttaaaatcagtggcggt-gccattaggctctaagcgtagtaaatctcct
cgctatagcg-tctggtggtcgctagccagaaccccaaaatttt---tatgattgacctc
ggatcaggtagggatacccgctgaacttaa------------------------------

>KF296960.1 Uncultured fungus


```
------------------------------------------------------------
------------------------------------------------------------
------------------------------------------------------------
------------------------------------------------------------
------------------------------------------------------------
-------------------------------------------------agtcgtaacaa
ggtttccgtaggtg-aacctgcggaaggatcattaccgagttcatgccct---tcggggt
agatctcccaccctttgtata-ctatacct--ctgttgctttggcgggcc--gtctagct
a--------ctggcttcggctggtaagtgcccgccaga-ggacccaaaccct-ga--a-
tat--tagtgtcgtctgagtaaaaag--tttaatatttaaactttcaacgac-ggatct
cttggctctggcatcgatgaagaacgcagcgaaatgcgataagtaatgtgaattgcttaa
ttcagtgaatcatcgaatctttgaacgcacattgcgccccttggtattccgaggggcatg
cctattcgagcgtcattatcacccctcaagc-ct-agcttggtgttgagacc-tgctgt-
taaggcagtctctaaaatcagtggcagt-gctgtcaggctctaagcgtagt-aaattcat
cgctatagggtcctggtggatactcgccaaaaccccccatttttt-aatgattgacctc
ggattaggtagggatacccgctgaacttaagcatatcaataagcggaggaaaagaaacca
acagggattgcctcagtaacggcgagtgaagcggcaaaagctcaaatttgaaatctggct
cttttagggtccgagattgtaatttgtagaagatggtttcgggtgtggctccggtttaag
-tctttggaatattacatcatagagggtgagaatcccgtatgtgaccggcagccttcgcc
tatgtgaaactctttcancgagtcgagttgtttgggaatgcagctcaaaatgggaggtat
atttcttctaaagctaaatattggccagagaccgatagcgcacaagtagagtgatcgaaa
gatgaaaagcactttggaaagagagttaaacagtacgtgaaattgttgaaagggaagcgc
ttgcacccagacttgcacgcagttgatcatccggtgttctcaccggggcactgtgctgcg
ttcaggccagcctcggttttggtggcaggataaaggccttgggaatgtggcttccttcgg
ggagtgttatagccctcggtgcaatgcagcccccgggaccgaggaccgcgcttcggcta
ggatgctggcgtaatggttttcagcgacc-------------------------------
------------------------------------------------------------
------------------------------------------------------------
------------------------------------------------------------
------------------------------------------------------------
------------------------------------------------------------
------------------------------------------------------------
------------------------------------------------------------
------------------------------------------------------------
------------------------------------------------------------
------------------------------------------------------------
------------------------------------------------------------
------------------------------------------------------------
------------------------------------------------------------
------------------------------------------------------------
------------------------------------------------------------
------------------------------------------------------------
------------------------------------------------------------
------------------------------------------------------------
------------------------------------------------------------
------------------------------------------------------------
------------------------------------------------------------
------------------------------------------------------------
------------------------------------------------------------
------------------------------------------------------------
------------------------------------------------------------
------------------------------------------------------------
------------------------------------------------------------
------------------------------------------------------------
```


>JX029127.1 Tetracladium sp. isolate

--atctcccaccctttgtata-ctatacct--ctgttgctttggcgggcc--gcctagct
a--------ctggcttcggctggtaagtgcccgccaga-ggacccaaaccct-ga--a-
tat--tagtgtcgtctgagtaaaaag--tttaatatttaaaactttcaacaac-ggatct
cttggctctggcatcgatgaagaacgcagcgaaatgcgataagtaatgtgaattgcagaa
ttcagtgaatcatcgaatctttgaacgcacattgcgccccttggtattccgaggggcatg
cctattcgagcgtcattatcaccccctcaagc-ct-agcttggtgttgagacc-tgctgt-
caaggcagtctctaaaatcagtggcagt-gctgtcaggctctaagcgtagt-aaattcat
cgctatagggtcctggtggatactcgtcaaaaccccccatttttt-aatgattgacctc
ggattaggtagggatacccgctgaacttaagcatatcaataa------------------

>MN660389.1 Uncultured Tetracladium sp.

-------------------------cgcacattgcgccccttggtattccgaggggcatg
cctattcgagcgtcattatcacccctcaagc-ct-agcttggtgttggggct-tgctgt-
aaaggcagcctctaaaatcagtggcagt-gctgtcaggctctaagcgtagt-aaattcat
cgctatagggtcctggtggatactcgtcaaaacccccc--attttt-aatgattgacctc
ggat--

>MG756632.1 Uncultured fungus

---------------------------------gaacgag---atgccttc----ggggc
ggatctccaaccctctgtata-ctacaattgtctgttgctttggcgggcccgcgcaagct
g--------ccggcttcggctggttagtgcccgccaga-ggaaactaaaccct-aac-aa
ttt--atgtttcgtctgaa-aaaata--ttaaatatttaaaactttcaacaac-ggatct
cttggctctggcatcgatgaagaacgcagcgaaatgcgataagtaatgtgaattgcagaa
ttcagtgaatcatcgaatctttgaacgcacattgcgccccttggtattccgaggggcatg
cctgttcgagcgtcattatcacccttcaagc-tt-tgcttggtgttggggca-tgccatc
acaggcagcccttaaaatcagtggcagtaaccattaggctctaagcgtagtaattcttct
cgctatagcg-tctggtggtcgcgcgccaaaaccccccattttt---catgattgacctc
ggatcaggtagggatacccgctgaacttaagcatatcaataagcggaggaaaagaaacca
acagggattgcctcagtaacggcgagtgaagcggcaaaagctcaaatttgaaatctggct
cttttagggtccgag-ttgtaatttgcagaagat-gctttgggtgtggctccagtttaag
ttctttggaatattacatcacagagggtgagaatcccgtatgtgactggctgccttcgcc
cgtgtaaagctctttcgacgagtcgagttgtttgggaatgcagctcaaaatgggtggtat
atttcatctaaagctaaatattggccagagaccgatagcgcacaagtagagtgatcgaaa
gatgaaaagcactttggaaagagagttaaacagtacgtgaaattgttgaaagggaagcgc
ttgcaaccagacttgcacgcagttgatcaaccggtgttctcaccggggcactctgctgcg
ttcaggccagcatcggttttggtggttggataaaggccttgggaatgtagcttctttcgg
ggagtgttatagcccaaggtgcaatgcagcctaccgggaccgaggaccgcgcttcggcta
ggatgctggcgtaatggttgtaagcggcccgtcttgaaacacggaccaaggagtctaaca
tctatgcgagtgtttgggtgtcaaacccatacgcgtaatgaaagtgaacggaggtaagaa
cccttcggggggcattatcgaccggtcctgatgtcttcggatggatctgagtaagagcat
agctgttgggacccgaaagatggtgaactatgcgtgaatagggtgaagccagaggaaact
ctggtggaggctcgcagcggttctgacgtgcaaatcgatcgtcaaa-ttgcgcatagggg
cgaaagac-tatcgaaccctccattcg---------------------------------

>MH451294.1 Uncultured fungus

-----------------------aacgcacattgcgccccttggtattccgaggggcatg
cctgttcgagcgtcattatcacccttcaagc-tt-tgcttggtgttggggca-tgccatc
acaggcagcccttaaaatcagtggcagtaaccattaggctctaagcgtagtaattcttct
cgctatagcg-tctggtggtcgcgcgccaaaaccccccattttt---catgattgacctc
ggatcaggtagggatacccgctgaacttaa------------------------------

>LR863329.1 uncultured Tetracladium sp.


```
------------------------------------------------------------
------------------------------------------------------------
------------------------------------------------------------
------------------------------------------------------------
------------------------------------------------------------
------------------------------------------------------------
------------------------------------------------------------
------------------------------------------------------------
------------------------------------------------------------
------------------------------------------------------------
------------------------------------------------------------
------------------------------------------------------------
------------------------------------------------------------
------------------------------------------------------------
------------------------------------------------------------
------------------------------------------------------------
------------------------------------------------------------
------------------------------------------------------------
------------------------------------------------------------
------------------------------------------------------------
------------------------------------------------------------
------------------------------------------------------------
------------------------------------------------------------
------------------------------------------------------------
-----------------------aacgcacattgcgccccttggtattccgaggggcatg
cctattcgagcgtcattatcacccctcaagc-ct-agcttggtgttgaggcc-tgctgt-
taaggcagcctctaaaatcagtggcagt-gctgtcaggctctaagcgtagt-aatttcat
cgctatagggtcctggtggatactcgccaaaacccccccatttttcaaatgattgacctc
ggattaggtagggatacccgctgaacttaa------------------------------
------------------------------------------------------------
------------------------------------------------------------
------------------------------------------------------------
------------------------------------------------------------
------------------------------------------------------------
------------------------------------------------------------
------------------------------------------------------------
------------------------------------------------------------
------------------------------------------------------------
------------------------------------------------------------
------------------------------------------------------------
------------------------------------------------------------
------------------------------------------------------------
------------------------------------------------------------
------------------------------------------------------------
------------------------------------------------------------
------------------------------------------------------------
------------------------------------------------------------
------------------------------------------------------------
------------------------------------------------------------
------------------------------------------------------------
------------------------------------------------------------
------------------------------------------------------------
------------------------------------------------------------
------------------------------------------------------------
```


>KX192428.1 Uncultured fungus


```
------------------------------------------------------------
------------------------------------------------------------
------------------------------------------------------------
------------------------------------------------------------
------------------------------gaaatgcgataagtaatgtgaattgcagaa
ttcagtgaatcatcgaatctttgaacgcacattgcgccccttggtattccgaggggcatg
cctattcgagcgtcattatcacccctcaagc-ct-agcttggtgttgaggcc-tgctgt-
taaggcagcctctaaaatcagtggcagt-gctgtcaggctctaagcgtagt-aatttcat
cgctatagggtcctggtggatactcgccaaaacccccccatttttt-aatgattgacctc
ggattaggtagggatacccgctgaacttaa------------------------------
------------------------------------------------------------
------------------------------------------------------------
------------------------------------------------------------
------------------------------------------------------------
------------------------------------------------------------
------------------------------------------------------------
------------------------------------------------------------
------------------------------------------------------------
------------------------------------------------------------
------------------------------------------------------------
------------------------------------------------------------
------------------------------------------------------------
------------------------------------------------------------
------------------------------------------------------------
------------------------------------------------------------
------------------------------------------------------------
------------------------------------------------------------
------------------------------------------------------------
------------------------------------------------------------
------------------------------------------------------------
------------------------------------------------------------
------------------------------------------------------------
------------------------------------------------------------
------------------------------------------------------------
------------------------------------------------------------
------------------------------------------------------------
------------------------------------------------------------
------------------------------------------------------------
------------------------------------------------------------
------------------------------------------------------------
------------------------------------------------------------
------------------------------------------------------------
------------------------------------------------------------
------------------------------------------------------------
------------------------------------------------------------
------------------------------------------------------------
------------------------------------------------------------
------------------------------------------------------------
------------------------------------------------------------
------------------------------------------------------------
------------------------------------------------------------
------------------------------------------------------------
------------------------------------------------------------
------------------------------------------------------------
```


>KM246272.1 Tetracladium sp.

-----------------------------------ttagaggaagtaaaagtcgtaacaa
ggtttccgtaggtg-aacctgcggaaggatcattaccgaagtataggcccc-tcgcgggc
tgaacttccaccctttgttta-ccatacct--ttgttgctttggcgggcc--cggcccct
aaagcccaccggccccaggctggtgagcgcccgccaga-gacccataaaccca-aaccat
tta--cagtgtcgtctgagaaaactt--ttaattatttaaaactttcaacaac-ggatct
cttggctctggcatcgatgaagaacgcagcgaaatgcgataagtaatgtgaattgcagaa
ttcagtgaatcatcgaatctttgaacgcacattgcgccccttggtattccgggggcatg
cctgttcgagcgtcattatcacccctcaagc-tc-agcttggtgttggggcc-tgctgta
aaaggcagcccttaaaatcagtggcggt-gctgtcaggctctaagcgtagtaacatatct
cgctatagggtcctggtggtgtcttgccaaaaccccacac-ttttt-aatgattgacctc
ggatcaggtagggatacccgctgaacttaagc----------------------------

>KM246209.1 Tetracladium sp.

---gaagtaaaagtcgtaacaa
ggtttccgtaggtg-aacctgcggaaggatcattaccgaagtataggcccc-tcgcgggc
tgaacttccaccctttgttta-ccatacct--ttgttgctttggcgggcc--cggcccct
aaagcccaccggccccaggctggtgagcgcccgccaga-gacccataaaccca-aaccat
tta--cagtgtcgtctgagaaaactt--ttaattatttaaaactttcaacaac-ggatct
cttggctctggcatcgatgaagaacgcagcgaaatgcgataagtaatgtgaattgcagaa
ttcagtgaatcatcgaatctttgaacgcacattgcgccccttggtattccggggggcatg
cctgttcgagcgtcattatcacccctcaagc-tc-agcttggtgttggggcc-tgctgta
aaaggcagcccttaaaatcagtggcggt-gctgtcaggctctaagcgtagtaacatatct
cgctatagggtcctggtggtgtcttgccaaaaccccacac-ttttt-aatgattgacctc
ggatcaggtagggatacccgctgaacttaa------------------------------

>MH871883.1 Botrytis cinerea strain CBS 261.71

-------------atggctaagtgaggctttcggactgccttagggagggtggcaacacc
cacccagaggcggaaagttatccaaacttggtcatttagaggaagtaaaagtcgtaacaa
ggtttccgtaggtg-aacctgcggaaggatcattacagagttcatgccc---gaaagggt
agacctcccaccc-ttgtgta-ttattact--ttgttgctttggcgagct----------
-----------gccttcgggccttgtatgctcgccaga-gaataccaaaactc-tt--tt

tat--taatgtcgtctgagtacta----tataatagttaaaactttcaacaac-ggatct
cttggttctggcatcgatgaagaacgcagcgaaatgcgataagtaatgtgaattgcagaa
ttcagtgaatcatcgaatctttgaacgcacattgcgccccttggtattccgggggcatg
cctgttcgagcgtcatt-tcaaccctcaagc-tt-agcttggtattgagtctatgtcagt
aatggcaggctctaaaatcagtggcggc-gccgctgggtcctgaacgtagtaatatctct
cgttacaggttctcggtgtgcttctgccaaaaccc--aaatttttc-tatggttgacctc
ggatcaggtagggatacccgctgaacttaagcatatcaataagcggaggaaaagaaacca
acagggattacctcagtaacggcgagtgaagcggtaaaagctcaaatttgaaatctggct
cttttagagtccgaa-ttgtaatttgtagaagat-gcttcgggtgtggttccggtctaag
ttccttggaacaggacgtcatagagggtgagaatcccgtatgtgactggatacctatgct
catgtgaagctctttcgacgagtcgagttgtttgggaatgcagctcaaaatgggaggtat
atttcttctaaagctaaatattggccagagaccgatagcgcacaagtagagtgatcgaaa
gatgaaaagcactttggaaagagagttaaacagtacgtgaaattgttgaaagggaagcgc
ttgcaatcagacttgcacttggtgttcatcagggtctcgtaccctgtgtacttcatcaag
ttcaggccagcatcagtttgagtggttagataaaggcttagagaatgtggccctcttcgg
ggggtgttatagctctaggtgcaatgtagcctacttggactgaggaccgcgcttcggcta
g---

